# Forecasting asylum-related migration flows with machine learning and data at scale

**DOI:** 10.1038/s41598-022-05241-8

**Published:** 2022-01-27

**Authors:** Marcello Carammia, Stefano Maria Iacus, Teddy Wilkin

**Affiliations:** 1 University of Catania, Via Vittorio Emanuele II, 49, 95125 Catania, CT Italy; 2 European Asylum Support Office (EASO), MTC Block A, Winemakers Wharf, Grand Harbour Valletta, 1917 MRS Malta; 3 European Commission, Joint Research Centre, Via Enrico Fermi, 2749, 21027 Ispra, VA Italy

**Keywords:** Computer science, Computational science, Scientific data, Statistics

## Abstract

The sudden and unexpected migration flows that reached Europe during the so-called ‘refugee crisis’ of 2015–2016 left governments unprepared, exposing significant shortcomings in the field of migration forecasting. Forecasting asylum-related migration is indeed problematic. Migration is a complex system, drivers are composite, measurement incorporates uncertainty, and most migration theories are either under-specified or hardly actionable. As a result, approaches to forecasting generally focus on specific migration flows, and the results are often inconsistent and difficult to generalise. Here we present an adaptive machine learning algorithm that integrates administrative statistics and non-traditional data sources at scale to effectively forecast asylum-related migration flows. We focus on asylum applications lodged in countries of the European Union (EU) by nationals of all countries of origin worldwide, but the same approach can be applied in any context provided adequate migration or asylum data are available. Uniquely, our approach (a) monitors drivers in countries of origin and destination to detect early onset change; (b) models individual country-to-country migration flows separately and on moving time windows; (c) estimates the effects of individual drivers, including lagged effects; (d) delivers forecasts of asylum applications up to four weeks ahead; (e) assesses how patterns of drivers shift over time to describe the functioning and change of migration systems. Our approach draws on migration theory and modelling, international protection, and data science to deliver what is, to our knowledge, the first comprehensive system for forecasting asylum applications based on adaptive models and data at scale. Importantly, this approach can be extended to forecast other social processes.

## Introduction

The 2015–2016 refugee crisis in Europe was sudden and unexpected. The humanitarian consequences were dire, with thousands of asylum seekers dead or missing in the journey^1^. The consequences in countries of destination also were significant. The actions taken by governments to uphold access to asylum procedures were generally reactive, uncoordinated and ineffective.

One important cause of the ineffective responses was a poor capacity to anticipate the movements of asylum seekers^2^. Forecasting asylum-related migration is indeed extremely problematic. Migration is a complex system^3^, which means that causal factors interact nonlinearly, are highly context dependent, and show little or no persistence over time. Potential drivers are diverse^4,5^, plus effect sizes and interactions vary widely between and within individual migration flows. In one context extreme conflict, violence and persecution may generate few asylum seekers; whereas elsewhere relatively subtle social unrest may spark large international displacements, particularly if they are a tipping point of deteriorating conditions. The effect of migration drivers is subject to threshold and feedback effects. Once activated, country to country flows tend to trigger self-reinforcing processes resulting in the establishment of migration systems^6–8^.

Migration is therefore a highly uncertain process^9^, which complicates migration modelling^10^. Among migration types, forced or asylum-related migration is associated with the highest uncertainty^9,11^. As a consequence, most quantitative asylum migration models focus on single drivers in countries of origin (e.g. conflicts^12–14^) or destination (e.g. migration or asylum policies^15–17^). Some more comprehensive asylum migration models have been developed, but these aim to increase retrospective understanding^12,18–22^ or provide alerts^23^ rather than forecasting flows, with exceptions mostly confined to the prediction of single country to country flows^24^.

Data on migration in general and its drivers also contain uncertainty, which further complicates migration modelling^25^. Despite recent advances in the collection of official statistics, particularly in the subfield of asylum, and in spite of the ongoing efforts to improve data collections at the international (notably in the European Union at Eurostat, the European Asylum Support Office (EASO, the European Union Agency for Asylum), the European Border and Coast Guard Agency (Frontex), and the European Commission's Knowledge Centre on Migration and Demography) and global (particularly at the International Organization for Migration and the United Nations Refugee Agency) levels, most data collections are limited in terms of frequency, definitions, coverage, accuracy, timeliness, and quality assurance^26–28^. This is also the case for data on migration drivers such as conflicts, the state of human rights and the economy—notably with regards to their frequency, accuracy and timeliness—all of which are prerequisites for effective forecasting.

Recent advances in data and computational technology, as well as the application of the methods of physics and complexity science to societal challenges^29,30^, are opening up new avenues for modelling, explaining and predicting social processes. Innovative data and computational approaches underpinned some progress in asylum migration modelling and forecasting. Large data sets containing vast reams of structured and unstructured data have been proposed as an opportunity to observe potential migration drivers as they occur in near to real time^31,32^. New data sources include mobile data^33^, social media^34,35^, and internet searches^36^. Big data are increasingly analysed with such techniques as agent-based modelling^37^ and machine learning^38^ to detect patterns and identify potential migration drivers that would otherwise go unnoticed. Such advances enabled the development of novel migration forecasting models, including for forced and asylum migration, with encouraging results in terms of reliability and timeliness which makes them potentially useful in operational scenarios^38,39^. However, to our knowledge even the most advanced models have been applied to a limited number of flows rather than generalised to the regional or global levels.

Here we demonstrate that adaptive, dynamic machine learning algorithms can integrate administrative and non-traditional data at scale to effectively capture early warning signals of asylum-related migration and deliver short-term forecasts of asylum applications from any country of origin to any European Union Member State (hereafter EU Member State refers to countries that exchanged asylum data with EASO, that is, 27 EU Member States plus Norway, Switzerland and the United Kingdom.)—and in principle to any country that collects data on asylum applications with adequate frequency. Our system combines a range of data on migration drivers and processes at different locations: events and internet searches in countries of origin and transit to capture migration drivers^5,40^ and intentions^36^; detections of irregular crossings at the EU external border; and asylum processes in countries of destination to capture potential feedback effects of asylum processes and practices on the choice of destinations^17,41^.

Our modelling approach is grounded on migration theory and modelling, data science, and international protection. Theories of migration broadly inform our choice of covariates, but the approach is data driven. Our dynamic models are able to adapt to single dyads of origin and destination countries, using rolling windows of past data to select the migration driver configurations relevant to each dyad in a given time period. By modelling country-to-country dyads separately rather than attempting to build a single asylum migration model, we are able to address one of the most severe constraints to migration modelling—that is, that migration processes connect origin and destination countries in complex systems whose functioning vary largely over space and time. By delivering what is, to our knowledge, the first comprehensive system for forecasting asylum applications in potentially any context in which adequate data are available, we hope to contribute to international protection research and ultimately to better policy based on early warning and preparedness.

## Design

### Strategy

Our early warning and forecasting system proceeds by (a) monitoring migration drivers in countries of origin and destination to detect change early onset; (b) estimating the effects of individual drivers, including lagged effects; on those bases, (c) assessing how patterns of drivers shift over time to describe the functioning and change of migration systems; and finally, (d) producing forecasts of asylum applications in countries of destination up to four weeks ahead.

To observe covariates of migration at different points of migration processes, we exploit three tiers of data: geolocated events and internet searches in countries of origin; detections of illegal crossing at the borders of the EU; and asylum recognition rates in countries of destination. We leverage the potential of non-traditional data sources to position the analysis as close as possible to migration drivers as they happen. To addresses the complexity of migration systems and drivers our approach models individual country-to-country migration flows separately. Moreover, models are trained on moving time windows to account for change over time even within individual flows.

We applied our method on a wide range of bilateral asylum flows from circa 200 countries of origin worldwide, to each EU Member State. In Supplementary Note 2 (Supplementary Figs. S1–S10) we report performance results for 70 country-to-country flows, generated by seven countries of origin (Afghanistan, Eritrea, Iraq, Nigeria, Syria, Turkey and Venezuela) and nine countries of destination (Austria, Belgium, Germany, Greece, Spain, France, Italy, The Netherlands and Sweden), plus the EU as a whole. As shown in Supplementary Table S1, our model largely outperforms benchmark models in nearly all cases. The selected flows represent a suitably large diversity on the variables analysed; but our method can be applied in any context, provided adequate migration or asylum data are available.

**Figure 1:**
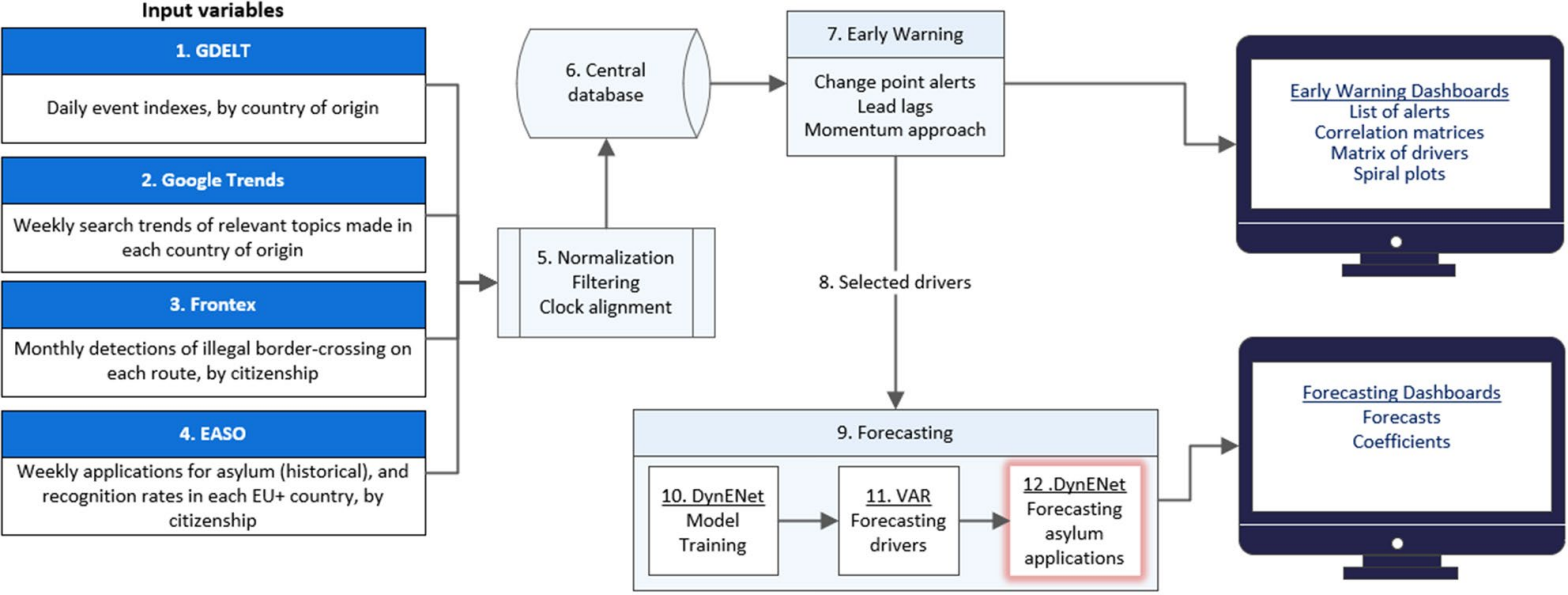
The Early Warning and Forecasting System workflow showing 4 categories of input variables (1–4), processing of datasets (5) storage (6), Early warning in the form of change alerts for each time series, lead lags, and correlation matrices (7), from which selected drivers (8) move to the forecasting sections (9) where they are trained (10), forecasted (11) in order to eventually forecast the outcome variable that is, applications for asylum (12).

The workflow of the early warning and forecasting system is sketched in Fig. 1.

#### Outcome variable

The outcome we predict is the number of asylum applications lodged in each country member of the EU Common European Asylum System (which includes EU Member States plus Norway and Switzerland, and is referred to as EU+), and in the EU+ as a whole [Fig. 1-(12)]. These data, broken down by nationality of each applicant, are shared with the European Asylum Support Office (EASO—the EU asylum agency) on a weekly basis. Although provisional, in 2019 these data underestimated asylum applications by just 6% at the EU+ level, compared to official national statistics compiled by Eurostat (EU 862/2007). Estimates of asylum applications typically have a maximum value imposed upon them by the capacity of the receiving country to react to sudden influxes and quickly register each application. This ceiling effect is demonstrated by fewer applications being registered during Christmas and Easter holidays due to the brief closure of some asylum offices, although applications tend to be registered shortly thereafter.

#### Covariates

To measure covariates, we combine administrative statistics with non-traditional data.

#### TIER1: events and internet searches in countries of origin

##### Events

We estimate the timing and location of ‘push factor’ events in nearly all countries of origin from the Global Database of Events, Language, and Tone (GDELT) project, [Fig. 1-(1)], a repository of 316 types of geolocated event reported in the world’s broadcast, print and web media, in 100 languages. We used GDELT 1.0 data, which are updated on a daily basis; GDELT 2.0 data are updated every 15 min and could potentially feed a real-time system. Single events can be covered multiple times by different media outlets around the world and therefore occur multiple times in the GDELT data, so we extract individual events from the overall media coverage and include each event only once in our data.

**Figure 2:**
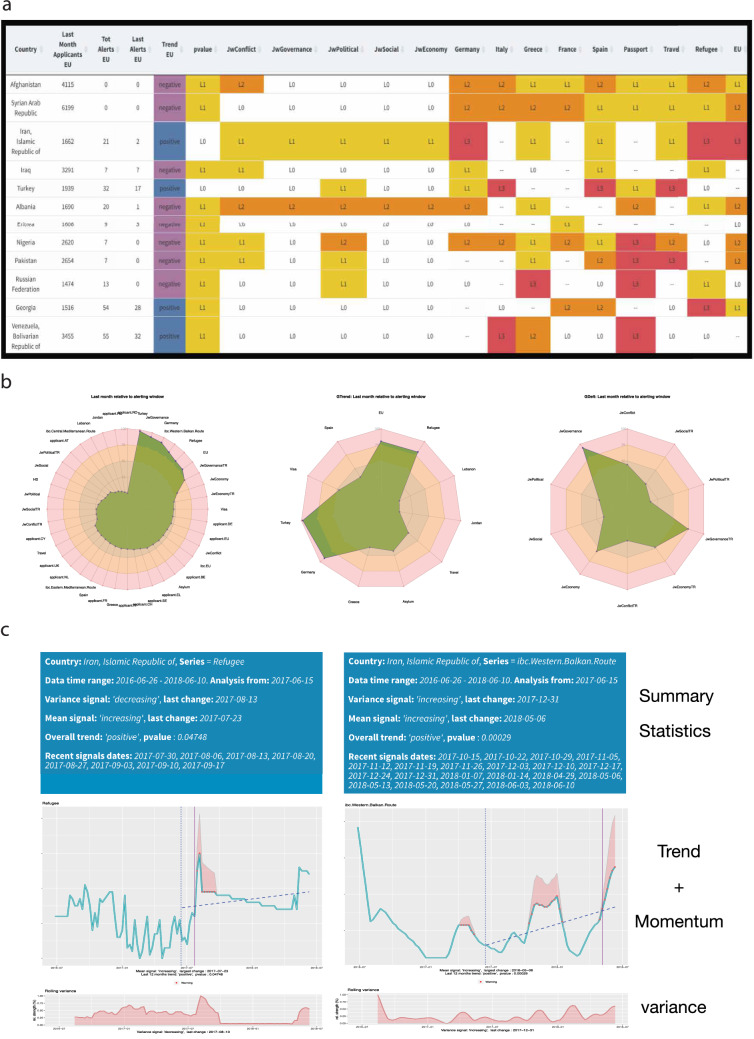

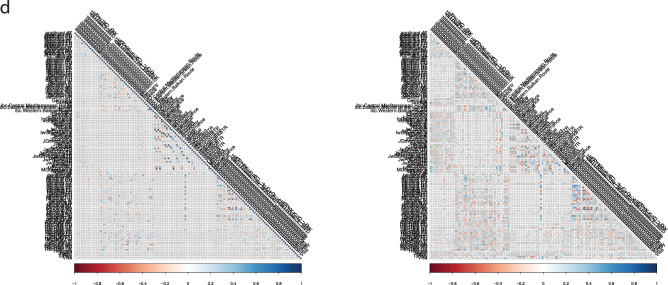
Early Warning Summary, week ending on 10/06/2018. (**a**) Early Warning Signals Table. Countries of origin included (rows): Afghanistan, Iran, Iraq, Albania, Eritrea, Georgia, Nigeria, Pakistan, Russia, Syria, Turkey, Venezuela. For each country, the table first shows the last month applicants in EU, the number of alerting signals observed and the trend of applications in the EU+ in the previous month. The degree of warning for each covariate (columns) is then shown: L0 (no warning) to L3 (max warning). Covariates included in the table are event macro-categories (conflicts, governance, political events, social unrest, economic events) and Google Trends topics [searches related to countries of destination (Germany, Italy, Greece, France, Spain, EU) and migration (passports, travel, refugee)]. The table identifies the time series that deserve closer inspection. (**b**) Iran, week ending on 10/06/2018. Radar plot of relative level of activity of single covariates in the early warning window, here set as one month, compared to the entire period of analysis: GDELT events and Google Trends searches, level during the early warning window relative to each series’ past values (left); Google Trends relative volume of searches (middle). GDELT event indexes relative level of activity (right). All series rescaled to 0–100%. (**c**) Iran, week ending on 10/06/2018. Time series with signals for individual covariates. In this figure: Google Trends searches for “Refugee” topic in Iran and Frontex’s “Illegal Border Crossings at the Western Balkan Route” of Iranian nationals. Top of each panel: the summary statistics, recent “momentum” signals, change point statistics for the mean and the variance. In the middle panel: data for the early warning windows with signals and change point analysis. Bottom panel: cumulative rolling variance to check for instability of the time series. (**d**) Iran, week ending on 10/06/2018. Correlation matrices, with (right quadrant) and without (left quadrant) shifting the time series for the optimal lag. At the optimal lag many correlation effects emerge, as shown by the increased density of the lagged correlation plot.

**Figure 3:**
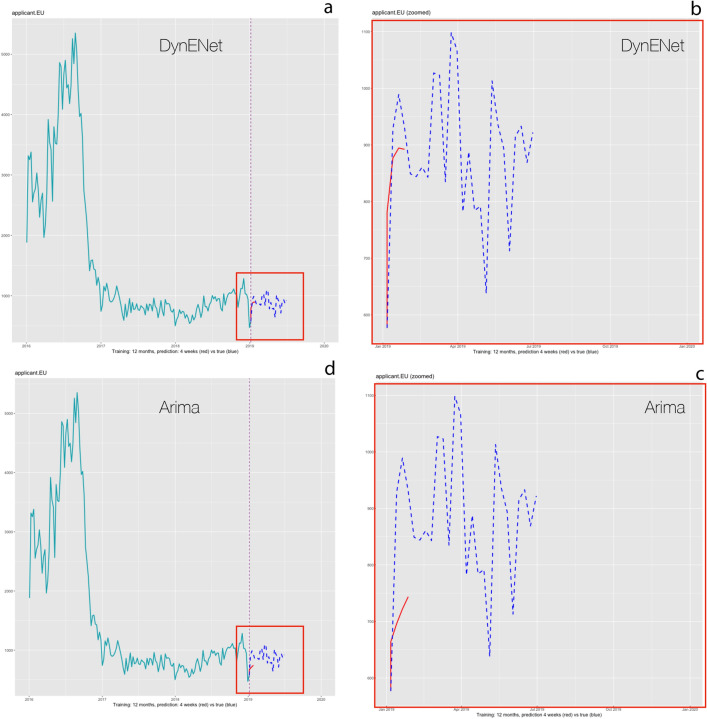
Forecast of applications by Afghan nationals in all EU+ for the four weeks following 30/12/2018. (**a,b**) DynENet model; (**c,d**) ARIMA model. (**a**,**c**) Show the full series, while (**b**,**d**) zoom in on the period starting with the forecast. Weeks are represented in the x axes, and the number of applications lodged by Afghan nationals in the y axes. The green line shows the number of applications lodged until the point in which the forecast is launched. The forecast is represented by the red line. The blue dotted line shows the actual number of applications lodged over the forecast period (and afterwards). The chosen week is a very difficult test for both models: the process has a huge drop down in coincidence with the end of the year when few applications were processed, and rebounds shortly after. The DynENet model copes better than ARIMA with the anomaly.

**Figure 4:**
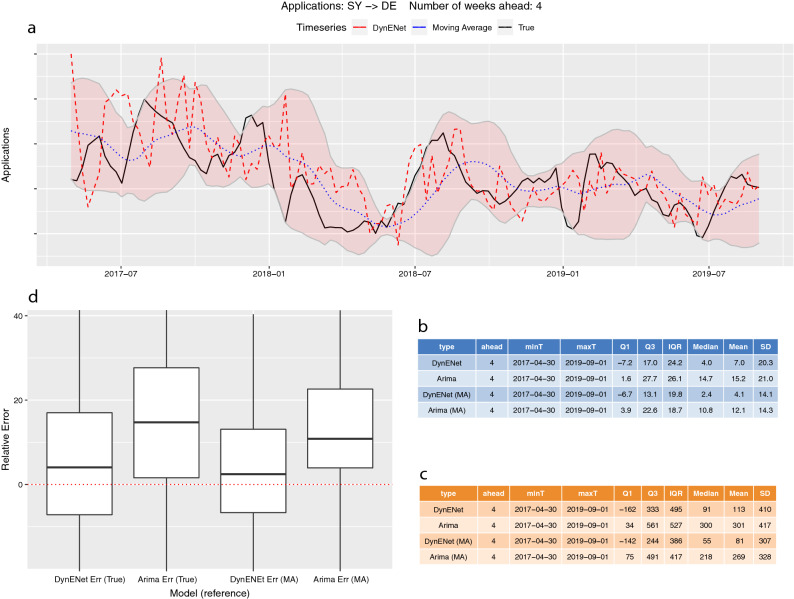
Back-testing performance of the system for forecasted applications by Syrians in Germany. (**a**) The black line shows the actual number of applications lodged by Syrian nationals in Germany. The dotted blue line is the moving average of the process. The red dashed line shows the DynENet 4-week ahead forecast at each time point. The pink shaded area represents a ± 2-standard errors confidence band around the moving average. (**b–d**) Summary statistics for the relative error (**b,d**) and for the absolute error (**c**). ARIMA, which is only based on the autocorrelation of the applications timeseries, is used as a benchmark model.

**Figure 5:**
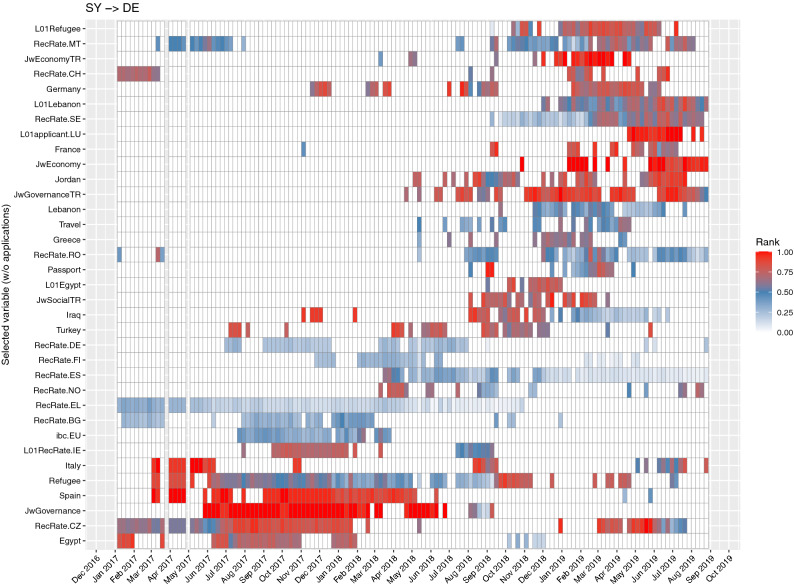
Predictors of asylum applications lodged by Syrian nationals in Germany in the period considered. The model adapts to the changing nature of the country-to-country dyad. The effect of some predictors is persistent in the first period observed (bottom-left); subsequently, the effect of those predictors fades and other predictors become important (upper right). The vertical axis shows all the predictors that have been selected by DynENet; the variables not shown were dropped by the forecasting model. The horizontal axis is the (weekly) timeline of the training period. Coloured cells denote the activation of given predictors at given weeks. The scale colour represents the relative importance of predictors evaluated through a Random Forest algorithm on the restricted model selected by DynENet, from 0 (white: predictor not selected in that week) to 1 (red: most important).

Not all events reported by the media are expected to be migration drivers and so we selected a subset of 240 events as potential drivers of migration and displacement^40^. Individual events differ in the extent to which they are likely to affect displacement and migration, therefore we weighted (±) each individual event according to its potential to generate displacement. Then we aggregated all weighted events into five macro-categories: *political events*, *social unrests*, *conflicts*, *economic events*, *governance-related events* [Fig. 1-(5)]. Finally, the total number of weighted events per macro-category per week were used in the following models, in order to align them with the frequency of the response variable. More details on event selection and weighting and on the general construction of event indexes are presented in the Methods section. The entire list of events and weights can be found as Supplementary Table S2.

##### Internet searches

Internet searches for particular topics may anticipate migration, and indeed internet searches have recently been used to estimate migration intentions and predict migration flows^36,42^. To estimate patterns of relevant internet searches, we use Google Trends, a publicly available tool providing multi-language, geolocated data on the relative frequency of search topics and terms [Fig. 1-(2)].

As shown in Table 1, we selected 17 topics (clusters of keywords) related to international migration and travelling in general (*visa* or *passport*), asylum seeking (*right of asylum* or *refugee*), countries of transit (e.g. *Jordan* or *Turkey* for searches that take place in Syria) and countries of destination (e.g. *Germany*, *France*, or *EU*). Then we downloaded the relative search frequencies for these topics in non-EU countries. The General User Interface of our system permits to easily customise the topic searches to include in the analysis of single countries. For example, we included searches related to some particular ‘transit countries’ among search topics (see Table 1). We selected those particular countries of transit for inclusion among topic searches because in the period covered by this analysis the arrivals were high on the Greek-Turkish border, and countries in the Middle East and Central Asia were the origin of a significant share of asylum applicants in Europe. Our model would then discard those variables if not relevant as predictors (for example, while performing forecasts of asylum applications by nationals of Venezuela). In principle, we could have included different sets of topic searches for each individual country of origin included in the analysis.

**Table 1:** Google trend topics (clusters of relevant keywords).

Migration/travel	Asylum	Transit	Destination
Passport	Refugee	Egypt	Cyprus
Travel	Right of Asylum	Iraq	France
Travel visa		Jordan	Germany
		Lebanon	Greece
		Turkey	Italy
			Spain
			European Union

#### TIER2: detections of irregular crossings at the EU external border

EU Member States and Schengen Associated Countries share monthly detections of illegal border-crossing with the European Border and Coast Guard Agency (Frontex) who make the data available on their website, aggregated at the level of ‘migration route’ (Eastern, Central, and Western Mediterranean; Western Balkans, Eastern Borders) by citizenship [Fig. 1-(3)]. Data are defined by EBGCA as “data reported on a monthly basis by Member States and Schengen Associated Countries on detections of illegal border-crossing on entry between Border Crossing Points of the external borders of the Member States of the EU and Schengen Associated Countries, and aggregated by routes”. [As EBCGA reports, “The data refer to detections of illegal border-crossing rather than the number of persons, as the same person may cross the external border several times. However, there is currently no EU system in place capable of tracing each person’s movements following an illegal border-crossing. Therefore, it is not possible to establish the precise number of persons who have illegally crossed the external border.” (See the notes to the data spreadsheet downloaded at https://frontex.europa.eu/along-eu-borders/migratory-map/, last checked on 20 October 2021)].

We are not aware of systematic research on the relationship between detections of illegal border-crossing and asylum applications (but see^15^). Analyses carried out at EASO suggest that they do covary but the strength of the relationship varies with nationality and location^43^. For example, detections of illegal border-crossing do not precede asylum applications for nationalities that can travel to the EU via regular means, i.e. with visa or under visa-free regimes, but they do precede asylum applications in locations where detection at the external border is inevitable, such as the Greek Aegean islands. In any case, we include detections of illegal border-crossing in all our models because our machine learning algorithm retains variables relevant to the individual flows and discards those that are not.

#### TIER 3: asylum processes in the EU+

To capture the potential effect on asylum applications of asylum processes and practice in countries of destination, we include data on ‘recognition rates’ [Fig. 1-(4)] calculated as the share of total asylum decisions that grant (rather than reject) international protection (Refugee status or Subsidiary protection status as defined in Article 2 of the Qualification Directive 2011/95/EU). EU Member States plus Norway and Switzerland share monthly asylum decisions with EASO broken down by the nationality of the applicant (validated data are later released by Eurostat). Recognition rates vary markedly between receiving countries, and they have been shown to be positively related to asylum applications^17^. Moreover, recognition rates can cause a deflection effects^15,18^ whereby lower recognition rates in one country may induce asylum seekers to lodge their applications in countries with higher recognition rates.

### Procedure

One of the most severe constraints to migration theory and modelling is that migration processes connect single country of origin to country of destination dyads in complex systems whose functioning vary largely over time and space. To address this constraint, rather than attempting to build a single asylum migration model we model each individual country-to-country dyad separately. In practice, the procedure starts with the selection of one country of origin (although the tool systematically goes through each country of origin) and proceeds with the analysis of time series on the three tiers of input variables:

1. In countries of origin:
  o events (5 macro-categories)
  p internet search queries (17 topics in the present implementation, although the system can accept any set of topics according to user choice)
2. At the external border of the EU:
  o detections of illegal border-crossing (across 4 migratory routes)
3. In countries of destination:
  o recognition rates in EU Member States, and in the EU+ as a whole
  p asylum applications in all EU+ countries, and in the EU+ as a whole

We also include events variables for third countries when relevant to certain migration routes. For example, Syrians and Afghans could pass through or stay in Turkey for some time before moving to, say, Germany; therefore, event variables for Turkey may have an effect on asylum applications by Syrians or Afghans in Germany. In general, the system is flexible in terms of selection of input variables.

Given that uncertainty increases with the forecasting horizon, we forecast the number of asylum applications (one, two, three, and) four weeks ahead. While a relatively short term, four weeks is a highly valuable time window for planning and preparedness purposes in an operational context.

We design a system that works in two steps: early warning and then forecasting (Fig. 1).

#### Early warning

In the first step—early warning analysis [Fig. 1 (7)]—for the focal country of origin, the system takes all input variables and detects signals of significant change by performing change point analysis for mean and variance in each time series. Then Pearson correlations are performed between all covariates and historical asylum applications lodged by nationals of the focal country of origin in the focal Member State, and the lags that maximise correlations between each pair of series are estimated.

This system is entirely data driven. The activation thresholds to trigger alerts depend on a moving average window of the latest data available. Single countries of origin have different “natural” levels of conflicts and other potential migration-generating events, different patterns of internet searches, generate different volumes of asylum applicants, and so forth. Fixed thresholds may result in inconsistent false positive alarms. The optimal predictive lag of each time series on the outcome variable—asylum applications—is found using a lead-lag estimation method^44^. In addition to lead-lag analysis and change point estimation, the acceleration of each time series is measured based on the ratio between shorter (6 weeks) and longer (24 weeks) moving averages (the idea being that if the short- and long-term moving averages are of the same order, the time series behave stably in the last 24 months, otherwise the series is accelerating/decelerating). This method is called ‘momentum approach’ in quantitative finance^45^ (but it is widely used in many disciplines). We set at ± 110% the ratio’s threshold for triggering alerts (cf.^46,47^), but like the other parameters of the early warning system this one also can be customised. The early warning analysis is explained in full details in the Methods section.

Figure 2 shows an illustrative early warning summary ran in the week ending on 10 June 2018 with a focus on the following countries of origin: Afghanistan, Iran, Iraq, Albania, Eritrea, Georgia, Nigeria, Pakistan, Russia, Syria, Turkey, Venezuela (and a closer focus on the particular case of Iran).

#### Forecasting

Also based on the signals generated in the early warning step (i.e., suggesting to the forecasting model those input variables that present unstable patterns), in the *forecasting* step [Fig. 1-(9)] the system estimates the future number of asylum applications in European countries of destination, aggregated by the nationality of the applicant. Our machine learning algorithm uses a rolling window of past data on single country-to-country dyads, including lagged covariates identified in the early warning step, to model those processes and then generate projections [Fig. 1-(8)].

More specifically, for each country-to-country dyad, the procedure consists in estimating a Dynamic Elastic Net Model (DynENet, see “Methods” section) on a moving time window of historical data. DynENet is the Elastic Net Method^48^ calibrated on a dynamic window. The procedure uses the 12 months preceding the observation point as a training set to estimate a DynENet model, as well as for further cross validation to minimise the Mean Squared Error (MSE) of the forecasts across the training period. Being an adaptive method that mixes LASSO-type^49^ (LASSO is a shrinking estimation method that drops uninformative input variables or, in case of multiple correlation, keeps only the most informative in the set of correlated input variables) and Ridge-type^50^ estimation (the Ridge method allows for estimation of models with correlated input variables, and instead of dropping some input variables it essentially returns an estimated model where their coefficients are averaged), the DynENet can take into account hundreds of variables for each dyad of country of origin and country of destination and it has the advantage of finding the most parsimonious model for each dyad – i.e., it performs model selection and estimation contextually. At the same time, the model also takes into account collinear variables as in Ridge regression [Fig. 1 (10–12)].

A VAR model is used to predict the future values of the input variables (see “Methods” section for details). For lagged variables, the real past values are considered as predictors [Fig. 1-(10)]. Finally, when all predictors have been forecasted, future values of the applications lodged at 1, 2, 3 and 4 weeks are obtained by feeding the forecasted predictors into the DynENet selected model [Fig. 1-(12)]. The optimal (see “Methods” section) DynENet model is then bench-marked against the ARIMA(1,0,1) model based solely on asylum applications.

The procedure for the forecasting step is explained in detail in the Methods section, and most parameters can easily be customised. By way of illustration, Fig. 3 shows a sample forecast for asylum applications lodged by nationals of Afghanistan in the EU+ in early 2019. The actual number of asylum applications is represented by the green line before the point when the forecast is simulated, and by the dotted blue line after that point. The four-week forecast is the red line.

Afghan nationals had been among the top-three nationalities for asylum applications in the EU+ for most of the time between 2014 and 2018. As was the case with most top-ranking nationalities, the volume of applications by Afghani nationals fell sharply after the EU and Turkey agreed to end irregular migration from Turkey—the main country of transit at that time—to the EU in March 2016 (See https://ec.europa.eu/commission/presscorner/detail/en/MEMO_16_963). As a result, detections of illegal border-crossings at the EU border by Afghan nationals went down from a monthly average of about 26,700 the year before, to about 1430 the year after. Asylum applications decreased markedly as a consequence, although the change in the asylum trend became visible some six months after the EU-Turkey statement. Between October 2015 and September 2016, the average number of monthly asylum applications lodged in the EU+ was 123,308, which went down to a monthly average of 63,170 between October 2016 and September 2017.

In addition to this structural change, the flow was also subject to cyclical movements and some seasonality—notably a drop at the end of each year when the processing capacity of asylum authorities tends to be severely limited, followed by subsequent increase. Both DynENet and ARIMA attempt to capture this behaviour, but DynENet is far more effective. In the four weeks considered in the back-test, the total number of applications was 3424; DynENet forecasted 3445 (0.6% relative error, 21 units absolute error) and ARIMA 2826 (− 17.5% relative error, 598 units).

## Forecasting performance

### Back-testing and forecasting accuracy

To test the forecasting performance, we simulated weekly forecasts from 30 April 2017 to 1 September 2019. The forecasting system took data from April 2016 to April 2017 and then iteratively moved onward by one week at every step. This means that the procedure replicated a hypothetical real forecast using only information that would have been available at each point in time, each time running early warning analyses to generate lagged variables that could be retained by the system in the forecasting step.

Figure 4 shows the back-testing results for an especially relevant flow: Syrian applicants (SY) in Germany (DE), effectively the largest flow in the EU+ for most of the time between 2014 and 2019. The series shows some typical patterns of asylum processes, such as non-regular cyclical oscillations, as well as some stylised properties of these administrative data such as the drop at the end of each year that has been observed above for Afghans applicants.

In almost all weeks, the forecast stays within the confidence bands of ± 2 standard errors, which means that the system performs statistically well. The exceptions are limited and occur mostly during the initial part of the analysis. That is largely due to the structural change in the series following the EU-Turkey statement in March 2016, which resulted in a radical change in the trend of asylum applications (see footnote 8 and Supplementary Note 1 for more details). Asylum-related migration can be a rather unstable process in general; including such a radical change within the training period makes the test extremely challenging. Our model typically adapts to change with a short delay; sometimes, for example around June 2018 and 2019, it manages to capture and anticipate abrupt changes. For the particular case of Syrians lodging applications in Germany, the average and median relative errors are 7% and 4% respectively (4.1% and 2.4% respectively from the moving average). For the benchmark ARIMA model, the average and median relative errors are 15.2% and 14.7%. Our model significantly outperforms the ARIMA model most of the time in most country-to-country flows (see Supplementary Note 1 for more details), which shows the added value compared to time series extrapolation methods based on autoregressive models.

Supplementary Note 2 shows the related figures for a selected sample of 10 additional flows: applications lodged by Syrian nationals in Greece, Sweden and the EU+; of Venezuelans in France, Spain and the EU+; of Nigerians in Germany and Italy; of Afghans in Germany (Supplementary Fig. S1-S10).

To further illustrate the performance of the system over time and space, in Supplementary Note 1 we present an analysis of forecasting performances for a selection of 70 dyads comprising of seven countries of origin (Afghanistan, Eritrea, Iraq, Nigeria, Syria, Turkey and Venezuela—together the source of 1,654,040 asylum applications in the EU+, or 47.6% out of a total of 3 473 050 applications received, between 2016 and 2019) and ten destinations (Austria, Belgium, Germany, Greece, Spain, France, Italy, The Netherlands and Sweden, and the EU+ as a whole). As discussed further in Supplementary Note 1, these dyads were selected to provide a large variation across relevant variables and thus test the performance of the forecasting system over largely different conditions.

More extensive analyses have been carried out. The data for extensive back-testing for the top-29 countries of origin of asylum applicants (Afghanistan, Albania, Algeria, Armenia, Bangladesh, Cameroon, China, Colombia, Côte d’Ivoire, Democratic Republic of Congo, Eritrea, Georgia, Guinea, Iran, Iraq, Libya, Macedonia, Morocco, Nigeria, Palestine, Pakistan, Russian Federation, Serbia, Somalia, Sudan, Syria, Turkey, Ukraine and Venezuela) are available upon request. These countries represented the origin of 81.5% of the applications lodged in the EU+ in the period analysed. However, the Early Warning and Forecasting System analyses all (circa 200) countries of origin and 30 EU+ countries of destination, and the same performance statistics can be generated for all country-of-origin-to-country-of-destination dyads.

### Adaptiveness of the DynENet model and assessment of change in migration systems

The matrix in Fig. 5 shows the variables selected by the DynENet model in each week of the back-testing period, still for the sample flow of Syrian applicants in Germany. Because the model is adaptive, the variables selected as well as their relative importance may vary from week to week. The relative rank is evaluated through a Random Forest algorithm on the restricted model selected by DynENet and represented through colours in Fig. 5—from 0 (“variable not included”) to 1 (“most important”). The colours therefore represent the relative importance of each predictor in the DynENet model.

The heatmap illustrates some key features of the model and of the underlying processes. First, the effects of single variables tend to persist for several weeks (horizontal colour bands). This indicates that country-to-country asylum flows have some (temporal) structure, which the model is able to capture. Variables are clustered in the heatmap in such a way to show this persistence. In Fig. 5, variables that were relevant in the first half of the observed time period are clustered in the lower left area, while variables relevant in the second part of the period are clustered in the upper right area.

The variables retained in the model describe the changing nature of the process, and therefore can be used to interpret it. For example, in the first part of the period observed, Syrian asylum applications in Germany were best predicted by such variables as the recognition rate in Greece, searches for Egypt- or refugee-related topics in Syria, or governance events in Syria. The process changed quite markedly in the summer of 2018, when the main predictors became internet searches for Greece, Germany, Iraq, Lebanon, passport- and travel-related topics, as well as lagged searches of Lebanon and refugee-related topics in Syria; economic events in Syria; social, economic or governance events in Turkey, a key transit country; and recognition rates in some other EU+ countries. In general, the variables selected by the model are largely consistent with the nature of single country-to-country flows, and their change over time.

Supplementary Note 2 shows the related figures for a selected sample of ten additional flows (see Supplementary Figs. S1–S10).

## Discussion

With increased numbers of displaced persons around the world, irregular migration and asylum have risen up the political agenda. If governments are to effectively manage mixed migration flows, they need to understand cause and effect and plan for future influxes. However, context dependent, short-lived complexity combined with sparse data means that forecasts are rare, tentative and unreliable.

This work takes a novel approach to align data on events and internet searches in countries of origin, detections of illegal border-crossing at the European border, and asylum decisions in European countries. These data are analysed by an adaptive machine learning system which delivers one- to four-week forecasts of mixed migration flows, consistently outperforming benchmark models. Rather than relying on a single migration model, the system adapts to the diversity of migration processes and drivers over time and space by training dynamic models on rolling windows of past data—separately for each country-to-country flow. This approach permits to address one major challenge to migration forecasting, that is, that migration processes connect origin and destination countries in complex systems whose functioning varies largely over space and time. Moreover, the results provide information on the specific causal factors relevant to each forecast, thus enabling a better understanding and analysis of mixed migration flows and their determinants. By delivering what is, to our knowledge, the first comprehensive system for effectively forecasting asylum applications in potentially any context in which adequate data are available, we hope to contribute to international protection and ultimately to better policy based on increased preparedness and resource allocation.

Drawing on migration theory and modelling, international protection, and data science, our approach delivers an effective system for early warning and forecasting of asylum related migration. But this approach has the potential to be adapted to understand and forecast other systems and processes characterised by complexity and sparse data.

## Methods

### Events indices

We extract from the GDELT database those events that occur in the lead paragraph of a document (coded as 1 on the “IsRootEvent” variable in GDELT). The GDELT project categorises events based on the CAMEO codebook, which includes 316 event categories. From the full list of events, we selected 240 categories that, according to theories of migration^4^ and ‘push factors’^40^, are most likely to represent potential drivers of migration^5^ (see Supplementary Note 3 and Supplementary Table S2). Because the potential to act as a driver varies across single events, we assigned a weight to each of the 240 different event topics, of the type *w* = pt/3, where pt is a number between − 4 and 4. The weight *w* is positive when the event is considered to potentially induce migration; *w* is negative when the event is considered to potentially reduce or constrain migration. We then aggregated single weighted events in five macro-categories: political events, social unrests, conflicts, economic events, governance-related events. The weights of each event within each macro-category are finally summed to obtain one index per macro-category. Having counted the number of unique (weighted) events for each day, we then aggregate them by week to align them with the frequency of the outcome variable (asylum applications): JwSocial, JwEconomics, JwPolitical, JwConflict, JwGovernance.

The CAMEO codebook includes some variables to account for the severity of events. One is QuadClass, which is used to classify events on a 1–4 scale (verbal cooperation, material cooperation, verbal conflict, material conflict). Another one is the Goldstein scale^51^, which assigns a numeric score ranging from − 10 to + 10 based on the theoretical potential impact of single events on country stability. Aggregated in a single composite indicator, that we call Push Factor Index (PFI)^52^, our indicator is strongly correlated to the negative Goldstein scale (0.9 Pearson correlation). We tested the correlation between yearly values of the PFI in 2016, 2017 and 2018, on the one hand, and recognition rates as well as one-year lagged asylum applications, on the other hand. On the average, Pearson correlation was respectively 0.54 and 0.47. Taking all events with a negative Goldstein scale, the average correlation with recognition rates and lagged asylum applications was slightly lower—respectively 0.51 and 0.42.

Because it is grounded on migration theory and should therefore reduce the level of noise in the indicators, and because it also seems more closely correlated to asylum applications and recognition rates, we prefer our event indexes to the available alternatives. The complete list of GDELT events and those selected for our indicators can be found in Supplementary Table S2.

### Internet search queries

We rely on the Google Trends tool to crawl the relative search of each topic (see Table 1) for all the countries in the world on a weekly basis.

### Forecasting procedure: early warning function

#### Statistical data cleaning and filtering

A preliminary analysis of each time series is performed in order to drop from the analysis any time series that has insufficient variability (i.e. no statistical information), which can be due either to too low values or to too many missing values as can occasionally be the case for applications and detections of illegal border-crossing (IBC). This stage of the pre-analysis has several filtering parameters as shown in Table 2. Before performing this task, the data are aligned to have a common weekly frequency. GDELT data have daily frequency and can be aggregated at the weekly level. Google Trends and asylum applications data are weekly. Data on illegal border- crossing and recognition rates have a monthly frequency and are transformed into weekly series through linear interpolation.

**Table 2:** Parameters for the early warning function.

Parameter	Default value	Details
country	No default value	Two digits ISO code for country of origin
cv.thr	0.05	Threshold on the coefficient of variation. Time series with coefficient of variation below the threshold are excluded from the analysis for this country
ibc.thr	100	IBC data threshold: if the maximal value of a specific IBC time series is below the threshold, the related data are dropped from the analysis
applicant.thr	100	EPS applicant data threshold: if the maximal value of a specific EPS applicant time series is below the threshold, the related data are dropped from the analysis
na.th	0.3	If any time series contains more than na.th*100 missing data, the time series is not reliable enough and hence dropped from the analysis
write.db	FALSE	Should write the result to a data base or on files? Currently, only FALSE is available, apart from a subset of data needed for forecasting which are stored anyway on the backend data base
refDate	Sys.Date()	The final date of the analysis
ma1	6	Length of the first moving average (in weeks)
ma2	24	Length of the second moving average (in weeks)
ma.th	1.1	Threshold of first and second moving average. If ma1/ma2 > ma.th, the signal is fired
clean.w	6	Data cleaning threshold, in months. All the dropping/cleaning pre-analysis is done only for the last window of data, i.e. the last 6 months. For example, if the maximal value of the IBC data in the last clean.w months is less than ibc.thr, the time series will be dropped
alert.w	12	Reference window to analyse the signals (in months)
back.w	24	Number of past months to consider in the analysis
pvalue	0.05	p-value threshold for assessing statistically significant structural change points in time series
llag.th	0.05	p-value threshold for assessing statistically significant lead-lag effects

The variability is taken into account in terms of the coefficient of variation (cv = standard deviation/|mean|). Being a pure number, i.e. without measure unit, it allows for variable independent thresholding: a time series whose variability is below 5% (the default value) is assumed to be statistically unreliable.

Time series of asylum applications and illegal border-crossing are also dropped if their maximal value in the month preceding the forecast is below the thresholds. We have different thresholds for applications and border crossings, as illustrated in Table 2.

#### Change point analysis

At this point the algorithm performs two change point tests: one for the mean of the time series and one for the volatility in the last 12 months. Statistical hypotheses testing on the change point are calculated with respect to the *p*-value parameter.

#### Spotting acceleration

Some characteristics of the time series could potentially complicate or bias the early warning analysis. For example, a time series may have a slope (positive or negative) without any statistically significant change point, or a change point may have occurred far in the past. The time series may also exhibit high variability and change points detected only due to isolated spikes, which however would not imply any persistent change. To take into account the variability or the speed of change, we compare a *short-* and *long*-term time series at MA1 = 6 months and MA2 = 24 months period. In a period of stationarity, those time series converge, but in case of positive (negative) acceleration, they diverge. If the MA1/MA2 is larger than a given threshold of 1.1, then a signal is fired by the early warning system. There might be isolated signals (the case of huge but isolated spikes) or a series of consecutive signals. In the latter case, the acceleration in activity/trend can be considered as a real signal. This technique is very well known in quantitative finance and called the *momentum approach*^36–38,45–47^ though used in many other disciplines.

#### Correlation and lead-lag analysis

Instead of analysing simultaneous correlations, Google Trends and GDELT data were analysed with a Lead-Lag approach, with one time series thereby anticipating another. The lead-lag effect is commonly noticed in financial econometrics. In time series analysis, this notion is considered a robust alternative to Granger causality (see^53^; for empirical evidence, cf.^54–57^), where a time series X is said to “Granger-cause” another series Y if past values of X provide statistical information on future values of Y, usually measured through a statistical t- or F-test in a VAR model specification. However, Granger-like approaches face several constraints: (1) the time series must be of the same frequency; (2) the time series must be linear (the model has to be a VAR one), and (3) testing for causality often leads to bidirectional effects. An additional problem is the Epps effect which states that, as the sampling frequency of time series increases, the empirical correlation is reduced^58^. The Lead-Lag approach overcomes the Epps effect by using Hoffman’s Lead-Lag estimator^44^ based on the Hayashi-Yoshida asynchronous covariance estimator^59–61^. This permits to apply a Lead-Lag approach to asynchronous, non-linear time series with different frequencies and missing data.

More precisely, let $\theta \in (-\delta ,\delta )$ be the time lag between the two nonlinear time series $X$ and $Y$. The approach consists in constructing a contrast function ${U}_{n}(\theta )$ which evaluates the Hayashi-Yoshida estimator for the times series ${X}_{t}$ and ${Y}_{t+\theta }$ and then to maximise it as a function of $\theta$. The lead-lag estimator ${\widehat{\theta }}_{n}$ of $\theta$ is defined as

$${\widehat{\theta }}_{n}=\mathit{arg} \underset{-\delta <\theta <+\delta }{\text{max}}|{U}_{n}(\theta )|.$$

When the value of ${\widehat{\theta }}_{n}$ is positive it means that ${X}_{t}$ and ${Y}_{t+{\widehat{\theta }}_{n}}$ (or ${X}_{t-{\widehat{\theta }}_{n}}$ and ${Y}_{t}$) are strongly correlated, therefore we say “$X$ leads $Y$ by an amount of time ${\widehat{\theta }}_{n}$”, so $X$ is the *leader* and $Y$ is the *lagger*. Vice versa for negative ${\widehat{\theta }}_{n}$. The assessment of the identified lag is done through a statistical test.

To retain the most significant lagged correlation effects, we also calculate the Lead-Lag-Ratio (LLR). LLR is used when there are two lead-lag effects, one for a positive lag and one for a negative lag, both statistically significant. In this case, the strongest among the two shortest (i.e., close to 0) lead-lag effects is returned by the LLR test statistics. Lead-lag and LLR as well as asynchronous correlation are available at present only through the yuima R package^62,63^.

### Forecasting procedure: forecasting function

The forecasting system attempts to forecast the value of the variable *applicants* from a country of origin (CoO) to different countries of destination (CoD) including the EU+ in aggregate. To this aim, the information from the early warning step is also used. This means that this task includes the dropping of some of the variables as explained above.

The analysis focuses on the variables named applicant.*, where * stands for one of CoD.

When data for “future” applications are available, as in back-testing, the system compares the forecast with the actual data. The complete list of arguments of the function forecast are illustrated in Table 3:

**Table 3:** Parameters for the forecast function.

Argument	Default value	Description
country	No default value	ISO 2 digit CoO country code
final.date	No default value	Should be in the format “YYYY-MM-DD”
start.date	"2017-01-01"	From where to start the back testing meta-analysis
n.ahead	4	Number of ahead periods prediction (in weeks)
prediction.win	12	Data used for the predictive model (in weeks)
alpha	0.5	ElasticNet parameter (see below)
burn	12	Number of data used in the local predictive statistical models (in weeks)

The forecasting model is the result of a meta-analysis based on all variables with lead-lag effects that entered and survived the previously exposed early warning step.

The forecasting strategy follows these steps:

- Set up a dynamic elastic net model
- Perform model estimation on a moving window of data
- Select the best (in terms of Mean Squared Error) dynamic elastic net model
- Forecast the covariates for the future periods exploiting also lagged variables
- Apply the estimated model to predict the outcome variable.

#### Dynamic elastic net model

Dynamic Elastic Net (DynENet) is a relatively new type of regularisation method which tries to perform model estimation and model selection in just one run.

Suppose we want to estimate a linear model of the form $y={\varvec{X}}\beta +\varepsilon$, where ${\varvec{X}}$ is the matrix of regressors which includes all the variables from the EWS analysis and $y$ is the dependent variable of interest (i.e., applications in this case). In this application, we have a huge number of regressors and relatively too few observations, which prevents us from estimating a new model. Regularisation methods, like DynENet, are also meant for dimensionality reduction, i.e., they estimate some of the beta coefficients as zero.

$$\underset{{\beta }_{0},\beta }{\text{min}}\left\{\frac{1}{N}\sum_{i=1}^{N}{w}_{i}l({y}_{i},{\beta }_{0}+{\beta }^{T}{x}_{i})+\frac{ \lambda }{2}\left[\left(1-\alpha \right)\left||\beta \right|{|}_{2}^{2}+2\alpha \left||\beta \right|{|}_{1}\right]\right\}$$

where, ${w}_{i}$ are weights for observations (${w}_{i}$=1 by default), $l(\cdot )$ is a loss function, normally the classical least squares contrast function, $\lambda$ is a penalty factor and $\alpha$ is a tuning parameter.

For $\lambda =0$, the formula becomes the usual LSE (least squares estimation) approach. For $\lambda =1$, this method becomes the so-called LASSO regression model, i.e., when trying to minimise the squared residuals from the model, the L1-penalty ($\left||\beta \right|{|}_{1}$ = sum of the absolute values of the regression coefficients) is added forcing some of the coefficients to be estimated as zero. Compared to a typical stepwise regression analysis, the LASSO procedure is not hierarchic and tries to select the best predictors in one step rather than estimating a sequence of nested models and selecting the one with the best R^2^ or AIC/BIC statistic. LASSO has been first introduced in genomics in the analysis of microarray data where the objective was to identify the set of genes (the input variables), out of a batch of thousands, that better describe the outcome (cancer) in an extremely small sample of patients. This method applies well to our setup of hundreds of input variables and not so long time series.

For $\alpha =0$, this model becomes the Ridge regression model, i.e. the classical regression with shrinkage for robust error estimation. For $\alpha =0.5$ the model is simply called Dynamic Elastic Net (DynENet). Using both L1 and L2 penalty at the same time is a good compromise in terms of prediction and interpretation. In fact, LASSO regression tends to keep only one among highly correlated subsets of regressors discarding all the others. With $\alpha =0.5$, the result of the regularisation takes into account also the correlation among the regressors and it results in a sort of “mean” effect of all variables that matter even though correlated among them. Notice that DynENet is also a variance shrinking method, which implies that the standard errors of the coefficients of the selected variables are relatively small compared to, e.g., linear regression.

The DynENet model itself has two tuning parameters: α and λ. The first one, α, is set to 0.5 in our approach, which means Lasso (L1-penalty) and Ridge (L2-penalty) estimations are equally weighted in the loss function of the optimisation problem. The parameter λ, the adaptive scaling factor for the penalties, is first estimated using cross-validation over a very large set of possible values in order to minimise the forecast MSE in the training data. This ‘optimal’ forecasting value is then used in the DynENet penalty function. This procedure is performed every week, so the optimal λ changes from week to week [Fig. 1-(10-12)].

The tuning parameter λ is quite important. The larger this number, the stronger DynENet will shrink the estimated coefficients to zero, in a potentially artificial way. To take into account this potential source of bias, the elastic net considers a grid of different values for λ and estimates the penalised regression model for a particular choice of α (in our case 0.5). Then λ is automatically selected ex-post by cross-validation.

In practice, what happens inside our forecast function is more complicated than the above classical DynENet algorithm. In particular, historical data on a time varying window are used to estimate the best DynENet model, i.e. the one with the cross-validated λ. Then the window is moved one week ahead, and the estimation procedure is calculated. This analysis is iterated until the current data available inside the forecast function. The final model, i.e. the final λ, is chosen among those models such that they attain the lowest prediction error (as measured by the mean squared error, MSE): for all models such that the estimated MSE is less or equal the variability of the time series, the optimal λ is extracted and then, the lowest λ among all lambda’s, is used in the subsequent analysis.

In summary, instead of considering the best model for the whole period of data, the forecast function selects the best model for each sub-period (time varying window) and then defines the optimal λ as the average and the minimal lambda in all the estimated models. This guarantees that the final forecasting model produces on average and in a robust way the best forecast in all periods. The optimal lambda shrinks the coefficients more than the minimal lambda.

#### VAR modelling

To obtain the forecast, the model also needs to simulate the future values of the covariates.

To this aim, a Vector Autoregression (VAR) model is first fitted on the historical values of the past 12 weeks of the covariates retained by the DynENet model; and then used to forecast future values of those covariates. When VAR model estimates do not converge, individual ARIMA(1,1) models for each predictor are ran. If the ARIMA(1,1) estimates fail to converge as well, the average value of the time series is considered. This multiple step approach is necessary because some of the time series may not be well approximated by stationary time series. For lagged variables, the real past values are considered as predictors [Fig. 1-(11)].

#### Sources of uncertainty

In practice there are two sources of approximation: the first is the simulation of the future values through a VAR/ARIMA/AR model and the second one is the prediction error of the estimated model on the historical data. Nevertheless, the forecasting models seem to be able to do a statistically sound job, in the sense that forecasts are generally within the 2 standard errors bands of the moving average process based on historical data (as seen e.g., in Fig. 4).

#### Random forest for variable ranking

To have an additional insight on the relative importance of each variable selected by the DynENet model, we run ex-post a random forest model and we rank the predictors according to the importance measure of this algorithm. As the number of predictors may vary from week to week, we consider the relative rank. This relative rank generates the colour scale in Fig. 5.

## Supplementary Information

Supplementary Information.

## Data Availability

The data that support the findings of this study are available from the authors. Restrictions apply to the availability of some of the data, and notably to weekly data on asylum applications and monthly data on recognition rates. Weekly asylum data are unvalidated and are exchanged for analytical purposes by the Member States of the EU Common European Asylum System under the Early Warning and Preparedness System of the European Asylum Support Office (for details, see https://easo.europa.eu/analysis-and-statistics). Validated data are made available by Eurostat normally two months after the reporting period, on the monthly level (see https://ec.europa.eu/eurostat/statistics-explained/index.php/Asylum_statistics). Weekly asylum data are not publicly available and were used under license for the current study. They may be available from the authors upon reasonable request and with permission of the national asylum authorities.

## Code availability

Source code for all the analyses and figures included in this paper are available from the authors for research and non-commercial use. R 4.0.0 language was used for the data cleaning, data mashup, statistical analysis and data visualisation. Several R packages have been used, in particular glmnet for the implementation of the Dynamic Elastic Net; yuima for the lead-lag analysis; sde, for the change point analysis as well as the package ts and VAR respectively for the ARIMA and VAR models estimation; randomForest was used to extract the relative importance of each predictor in the model selected by the Dynamic Elastic Net algorithm. The rest of the analysis was produced via own written R code.
