# Supplementary material for: Forecasting asylum-related migration flows with machine learning and data at scale
Source: Sci Rep. 2022 Jan 27;12:1457. doi: 10.1038/s41598-022-05241-8 (PMC8795256; doi:10.1038/s41598-022-05241-8)
Supplement: Supplementary file 1 — Supplementary Information. [file 41598_2022_5241_MOESM1_ESM.pdf]

Supplementary information for “Forecasting asylum-related migration flows with machine learning and data at scale”

Marcello Carammia[1,2,*], Stefano Iacus[3], Teddy Wilkin[2]

[1] University of Catania. Via Vittorio Emanuele II, 49, 95125 Catania (CT), Italy.
[2] European Asylum Support Office (EASO). MTC Block A, Winemakers Wharf. Grand Harbour Valletta, MRS 1917, Malta.
[3] European Commission, Joint Research Centre. Via Enrico Fermi, 2749, 21027 Ispra (VA), Italy.

* Corresponding author.

Contents

Supplementary Note 1. Back-testing. Forecasting performance on selected country-of-origin-to-country-of-destination flows
- Selection of countries and flows for back-testing
- Expectations
- Results
- Supplementary Table S1. Back-tested forecasting performance on selected representative flows. Summary statistics and comparison between DynENet model and benchmark ARIMA model.

Supplementary Note 2. Summary performance results on sample of flows
- Fig. S1. Back-testing performance of the system for forecasted applications by Syrians in Greece.
- Fig. S2. Back-testing performance of the system for forecasted applications by Syrians in Italy.
- Fig. S3. Back-testing performance of the system for forecasted applications by Syrians in Sweden.
- Fig. S4. Back-testing performance of the system for forecasted applications by Syrians in the EU+.
- Fig. S5. Back-testing performance of the system for forecasted applications by Venezuelans in France.
- Fig. S6. Back-testing performance of the system for forecasted applications by Venezuelans in Spain.
- Fig. S7. Back-testing performance of the system for forecasted applications by Venezuelans in the EU+.
- Fig. S8. Back-testing performance of the system for forecasted applications by Nigerians in Germany.
- Fig. S9. Back-testing performance of the system for forecasted applications by Nigerians in Italy.
- Fig. S10. Back-testing performance of the system for forecasted applications by Afghans in Germany.

Supplementary Note 3. Event categories: selection and transformation
- Supplementary Table S2. Event categories within the GDELT-CAMEO event codebook, and details on the construction of the event indices for the purpose of this study.

Supplementary Note 1. Back-testing. Forecasting performance on selected country-of-origin-to-country-of-destination flows

We tested the performance of the system by simulating forecasts over a period of 118 weeks from 30 April 2017 to 1 September 2019, with the training period starting from April 2016 to allow at least a 50-week training set. The system starts by analysing data from April 2016 to April 2017, and then moves onward by one week at every step. The procedure replicates a hypothetical real forecast, that is, only information that would be available at each point in time is used; at each iteration, early warning analyses are ran to generate lagged variables that can be retained by the system in the forecasting step.

Note that the period selected for the back-test was in fact a particularly challenging period for the forecasting system to perform well. This is because, in March 2016, the EU and Turkey agreed to severely curtail irregular migration across the Greek-Turkish border (See https://ec.europa.eu/commission/presscorner/detail/en/MEMO_16_963). What the EU calls the 'Eastern Mediterranean route' was one of the main access points for mixed migration flows to Europe. Between April 2015 and March 2016, detections of illegal border-crossing were 85433 on the monthly average. In March 2016, after the EU-Turkey statement, there were 27343, and in April 2016 they went down to just 4324. Between April 2016 and March 2017, there were on the average 2864 detections of irregular border-crossing on the Eastern Mediterranean route every month (Frontex data available at https://frontex.europa.eu/along-eu-borders/migratory-map/, last consulted on 1 July2020).

Because of the lag between detections of illegal border-crossing and the lodging of asylum applications, also due to the large backlog and delays that European asylum authorities were facing at that time, the level of applications went down a few months later. But it did so markedly: on the average, between October 2015 and September 2015, 123308 applications for asylum were lodged every month in EU countries; almost halved to an average of 63663 between October 2016 and

September 2017. Thus, the period between 2016 and 2017 includes a radical change in the magnitude of mixed migration flows to the EU+ but not necessarily a change in push factors in countries of origin which may have even increased. Hence, to include that period in the back-testing makes the test rather conservative.

Selection of countries and flows for back-testing

The early warning and forecasting system runs for all pairs of countries of origin (circa 200) to countries of destination (28EU Member States plus Norway and Switzerland at the time of the analysis), which amounts to a total of circa 6000 dyads or country to country flows. Here we report results of back-testing on a selection of 70 dyads, generated by seven countries of origin (Afghanistan [AF], Eritrea [ER], Iraq [IQ], Nigeria [NG], Syria [SY], Turkey [TR] and Venezuela [VE]) and nine countries of destination (Austria [AT], Belgium [BE], Germany [DE], Greece [EL], Spain [ES], France [FR], Italy [IT], The Netherlands [NL] and Sweden [SE]); plus the EU+ as a whole.

The cases selected represent a suitably large diversity on the variables analysed based on the following criteria (For a general overview of asylum trends, including background on countries of origin and processes and decisions in countries of destination, see the EASO's Annual Asylum Reports, lastly https://easo.europa.eu/asylum-report-2020)

Countries of origin

- Global region and distance from countries of destination:
    - Countries in the Middle East (SY and TR), Asia (AF and PK), Africa (NG and ER) and America (VE)
- Internal situation during the period analysed:
    - Ongoing civil war (SY)
    - High (AF, IQ) to medium (PK, TR) fragility/instability
    - Authoritarian regime (ER)
    - Economic crises with relatively more limited incidence of conflicts events (NG, VE)

- Average rates of recognition of international protection in Europe [Source: Eurostat, https://ec.europa.eu/eurostat/databrowser/view/MIGR_ASYDCFSTQ__custom_141866/default/table?lang=en (last consulted on 30 October 2020). Rates of recognition are calculated on the sum of all decisions taken in EU-28 countries in the period concerned by the test. “Positive decisions” are the share of decisions awarding refugee status according to the Geneva Convention or Humanitarian status on the number of overall decisions. “Including national forms of protection” means that national (that is, not regulated by the EU) forms of international protection are also counted among positive decisions]:
    - Higher: ER (42.4% positive decisions; 44.7 including national forms of protection); SY (88.6% positive decisions; 89.7% including national forms of protection)
    - Medium: AF (35.8% positive decisions; 47.3% including national forms of protection); IQ (44.1% positive decisions; 47.6% including national forms of protection); VE (10.7% positive decisions; 77.9% including national forms of protection); TR (42.4% positive decisions; 44.7% including national forms of protection) [Note that here we list Venezuelans among ‘medium-level’ rates of international protection countries because of the large gap between EU-regulated and national forms of protection awarded to Venezuelans nationals. This is clearly arbitrary as they could also be listed among ‘lower’ or ‘higher’ (or both), depending on what figures are taken into account. Our general purpose here, however, is only to show that there is high variation in the recognition rates of applications lodged by nationals of the set of countries included in the analysis]
    - Lower: NG (9.5%; 20% including national forms of protection).
- Internet penetration (Internet users: percentage, position in world rank – see https://en.wikipedia.org/wiki/List_of_countries_by_number_of_Internet_users)
    - Medium-high (TR 65%, 92; VE 64%, 94)
    - Medium-low (IQ 49%, 127)

    - Low or very low (SY 34%, 153; NG 27%, 167; AF 199, 11%)
    - World's lowest (ER 1%, 215)
- Outflows of asylum applicants to EU over the period analysed
    - Consistently high levels (among top-3 to top-5 countries of origin): SY, IQ, AF
    - Shifting from low-ranking to top-3: VE
    - Occasionally high throughout the period: NG
    - Relevant change: TR (never a top country of origin but significantly high for some time)
    - Consistently lower than the other countries of origin included: ER

Moreover, Turkey is contiguous with the EU via Greece and Bulgaria. In addition to being a relevant country of origin for part of the period, Turkey has been a major transit country for migration to Europe for most of the period used for training the model, as well as for the second part of the period analysed. It is currently the country hosting the most refugees in the world.

Countries of destination

- Geographic position:
    - On the external border of the EU (EL/Eastern Mediterranean, ES/Western Mediterranean, IT/Central Mediterranean)
    - Away from the external border, by increasing distance (AT, FR, DE, BE, NL, SE)
    - Southern Europe (EL, ES, FR, IT), Central Europe (AT, DE), Northern Europe (BE, NL), Scandinavia (SE)
- Institutions and policy regimes for dealing with asylum-related migration
    - Established (DE, FR, BE, NL, SE)
    - Recent (EL, ES, IT)
- Asylum inflows over the period analysed

- Consistently in the top-five countries of destination: DE, EL, FR, IT
- From relatively low-ranking to top-3 country of destination: ES
- High levels relative to size (per-capita applications level): SE, BE, NL, AT (for some time)

Flows

- Level, variation, volatility, and patterns of asylum inflows:

  High level throughout the whole period (AF, IQ, PK, SY)

  - More (AF, IQ, PK) or relatively less (SY) variation
  - Higher- (PK) or lower- (AF, IQ, SY) frequency change
  - High in the first (ER, NG) or second (TR, VE) part of the observed period

Expectations

The high diversity represented in the sample of cases selected for back-testing provides a useful combination for assessing the adaptive capacity of the model. For example, events might be expected to be useful predictors of applications by nationals of such a country as Syria that has been in a civil war for years; or of Afghanistan, although to a lesser extent. Events should also be relevant to predicting asylum flows from countries that were unstable although not plagued by civil wars, like Pakistan; as well as from Nigeria and Venezuela afflicted by severe economic crises. Key transit countries can provide powerful barriers against migration to Europe, and in the back-testing we have included among covariates events in Turkey to account for that.

Internet searches, in turn, may be relevant to predicting migration from a range of countries (Bohme et al 2020), but less so from countries where internet penetration is limited. For some extreme cases like Eritrea, which is a stable dictatorship where the population has barely any access to the internet, events and internet searches are expected to be poor predictors. Our model, however, also includes variables related to EU border crossing as well as policy processes and outputs in countries

of destination, such as applications and recognition rates in EU+ countries. These should be useful to support predictions when events or internet searches are not good predictors.

In general, the complexity of migration systems means that we cannot have strong expectations about the predictors of individual asylum flows. Even where we can conjecture about the relevance of individual factors to a given country to country flow, we still expect the system to change over time – and predictors to change accordingly. Therefore, we can only have some very general expectations about to the functioning and performance of the early warning and forecasting system, that we can group as follows:

1) Expectations related to the predictive capacity of the system:

- E1. The system should be able to predict asylum applications under different contexts
- E2. The forecasting error should normally stay within an acceptable confidence interval, i.e. +-2 standard errors from the real trend
- E3. Forecasts of relatively more stable, more patterned, and less volatile trends should have smaller errors than forecasts of more unstable, less patterned, and more volatile trends
- E4. The DynENet adaptive model should outperform a benchmark ARIMA model based on extrapolation from the trend

2) Expectations related to the capacity of the system to adapt to changing patterns:

- E5. Even in cases of unstable or volatile flows, the system should manage to quickly adapt to the trend.
- E6. In order to adapt to the functioning of individual migration systems, the system will select certain predictors and discard others. The selected predictors will vary across flows, as well as over time within the same flows to adapt to their dynamics.

Results

Table A1. shows summary statistics describing the performance of the system over the entire back-testing period and across the selected country of origin to country of destination dyads and the related

asylum flows. For each dyad, we show the relative and absolute average forecasting error. A1.1 includes results for the DynENet model, while A1.2 includes results for the benchmark ARIMA model. To make sense of the results, we use a simple traffic light system, with colours indicating the forecasting performance ranging from very good (light green), to good (dark green), poor (orange) and very poor (red) performance. Moreover, a blue shading is added to those instances in which ARIMA outperforms DynENet.

The ranges are based on a combination of relative and absolute average errors. We report both average and absolute errors because some high relative errors may still be very acceptable if absolute errors are small. For example, the 'very good performance' in the table includes those forecasts that have an average relative error smaller than 10% or an average absolute error smaller than 10. Because the forecasts are over a four-week horizon, an absolute error of 10 translates into just 2.5 asylum applications per week, on average. The double-thresholds are especially relevant to those flows that have a small magnitude, which means that forecasts can have a high relative but small absolute error. For similar reasons, flows of very small magnitude simply confound the assessment of the forecasting performance. Therefore, while the table shows all results for the sample of flows analysed, very small flows are marked grey to signal the limited or null significance to the testing exercise.

In addition to country-to-country flows, table A1 also includes flows from single countries of origin to the EU. We cannot use the same ranges designed for country-level flows to assess the performance of the system in forecasting EU-level asylum applications, therefore for the EU level we focus on relative errors only. All EU-level forecasts are very good except for the forecast of applications from Eritrean citizens, that is classified as good (but it only misses the 'very good' threshold by 0.3 points). Absolute errors for EU-level flows range between 63 and 267 (although the upper level is indeed an outlier, as the second-higher error is 149). When we divide those absolute errors by 30 EU+ countries, they become very low. Even if we divide absolute EU-level errors by 15 to account for the concentrated distribution of asylum applications across EU countries, absolute

errors are still small. In any case, this is a theoretical exercise because errors related to single countries are computed separately.

When we exclude EU-level forecasts and the three cases where the numbers were not significant enough to produce any forecast, we are left with 59 country of origin to country of destination dyads. Of those, 16 still have very small magnitude and are marked grey. Therefore, we can comment on 43 dyads. Out of them, our DynENet model performs very well in 30 cases, well in four cases, poorly in three cases, and very poorly in six cases. In other words, within our (highly diverse) sample, seven out of ten DynENet forecasts are very good, and eight out of ten are very good or good; two out of ten are poor or very poor.

In comparison, the ARIMA model performs considerably worse (although perhaps still acceptably). Out of 43 dyads, the ARIMA produces 11 very good forecasts and 12 good forecasts. Poor and very poor forecasts are eight each. In all but four cases out of 43 (or in 90% of the observed cases) DynENet outperforms the ARIMA model. Of the four dyads in which ARIMA performs better, one (Eritreans to Italy) is very poor with both models, and one (Syrians to The Netherlands) is very good with both models; therefore, we can consider the improvement of ARIMA on DynENet as non-significant. Significant improvements can be seen with the forecasts of Syrians to Spain and Iraqis to Greece – respectively, from very poor to poor and from poor to good.

DynENet

	Countries of destination																			
	Relative	Absolute	Relative	Absolute	Relative	Absolute	Relative	Absolute	Relative	Absolute	Relative	Absolute	Relative	Absolute	Relative	Absolute	Relative	Absolute	Relative	Absolute
	AT	AT	BE	BE	DE	DE	EL	EL	ES	ES	FR	FR	IT	IT	NL	NL	SE	SE	EU	EU
**SY**	**14,1**	**38**	**3,6**	**-7**	**7**	**113**	**13,2**	**31**	**47,5**	**60**	**18,8**	**45**	**75,2**	**11**	**-2,7**	**-12**	**13,7**	**20**	**5,6**	**267**
**IQ**	**20**	**9**	**6,2**	**7**	**1,5**	**-6**	**11,3**	**28**	17,3	-1	**9,5**	**3**	**15,6**	**8**	**8,5**	**3**	**13,2**	**13**	**5,9**	**149**
**AF**	**8,5**	**8**	**2,1**	**-1**	**15**	**118**	**8,9**	**35**			**-2,4**	**-28**	**15,8**	**6**	**11,6**	**2**	**12,9**	**7**	**4,4**	**107**
**NG**	**18,8**	**7**	13,9	0	**3,2**	**4**	12,9	0	36,3	5	**-0,5**	**-4**	**26,9**	**134**	**-3,8**	**-6**	14	2	**5,5**	**67**
**VE**			28,3	2	**-1,4**	**-3**			**-6**	**-214**	**-1,9**	**-3**	**1,5**	**-1**	7,5	-1	42,8	3	**-2,9**	**-101**
**TR**	20,5	2	14,6	1	**4,4**	**24**	**8,4**	**-1**	44,1	5	**-2,3**	**-11**	13,6	1	**1,1**	**-1**	**5,6**	**0**	**-2**	**-63**
**ER**	31,7	1	22,8	0	**16,3**	**42**	50,5	5	46,1	1	**1,9**	**-1**	**100,8**	**23**	**10,2**	**3**	41,1	2	**10,3**	**109**
Mean	13,8	17,66667	3,966667	-0,33333	6,571429	41,71429	10,45	23,25	20,75	-77	3,3	0,142857	39,3	30,16667	4,15	-1,83333	16,75	7,333333	3,828571	76,42857
SD	5,156549	17,61628	2,074448	7,023769	6,723272	53,09336	2,227854	16,41899	37,83021	193,7473	8,020598	22,22932	39,41472	51,472	6,789035	6,047038	12,34127	7,788881	4,684625	125,5586
Max	18,8	38	6,2	7	16,3	118	13,2	35	47,5	60	18,8	45	100,8	134	11,6	12	41,1	20	10,3	267
Min	8,5	7	2,1	1	1,4	3	8,4	-1	5	1	0,5	3	1,5	1	2,7	1	5,6	2	2,9	67

ARIMA

	Countries of destination																			
	Relative	Absolute	Relative	Absolute	Relative	Absolute	Relative	Absolute	Relative	Absolute	Relative	Absolute	Relative	Absolute	Relative	Absolute	Relative	Absolute	Relative	Absolute
	AT	AT	BE	BE	DE	DE	EL	EL	ES	ES	FR	FR	IT	IT	NL	NL	SE	SE	EU	EU
**SY**	14,3	37	6,4	0	15,2	301	18,5	80	29,2	-11	24,6	58	131,6	24	**-1,9**	**-10**	18,2	31	8,4	415
**IQ**	29,3	12	1,3	1	10,9	98	5,5	-12	40,2	0	10,6	2	15,4	6	2	-7	15	14	7,6	199
**AF**	16,3	27	-2,9	-12	17	129	-5	-77			-6,3	-70	16,8	6	14	4	17,3	13	4,8	115
**NG**	22,5	11	-6,1	-2	-2	-49	9,6	0	20,2	-1	-7,1	-24	26,4	99	-27,5	-51	4,5	0	6,3	81
**VE**			-2,7	-5	-8,3	-6			-13,3	-296	-8,4	-7	-8,9	-10	-12	-4	2,5	-1	-11	-262
**TR**	27,1	3	36,1	7	-0,9	-33	-0,9	-26	23,1	0	-8,3	-24	4	-2	-15,7	-21	23,1	6	-9,5	-172
**ER**	29,4	1	8,3	-10	23,2	51	43,2	3	33,7	1	1,6	-3	**189,6**	**56**	21,9	11	52,4	4	8,1	63
Mean	17,7	25	1,6	-3,66667	7,871429	70,14286	4,525	-8,75	7,95	-153,5	0,957143	-9,71429	61,81667	30,16667	-1,2	-12,3333	21,75	11,33333	2,1	62,71429
SD	4,275512	13,11488	4,657252	7,234178	11,66958	121,4721	10,26982	65,42871	30,05204	201,5254	12,53806	38,33499	79,5418	40,55819	18,31568	21,97878	16,22822	11,02119	8,534635	225,9341
Max	22,5	37	6,4	12	23,2	301	18,5	80	29,2	296	24,6	70	189,6	99	27,5	51	52,4	31	11	415
Min	14,3	11	1,3	0	2	6	5	0	13,3	0	6,3	2	8,9	6	2	4	4,5	0	4,8	81

Traffic light system legend

Very good		Rel error < 10% and/or abs error < 10
Good		Rel error < 10 and abs error > 20; OR rel err > 10 and abs < 20
Poor		Rel error > 10% and Abs error > 20 and < 40
Very poor		Rel error > 10% and Abs error > 40
Arima better		Arima better than Ada-ENet

Supplementary Table S1. Back-tested forecasting performance on selected representative flows. Summary statistics and comparison between DynENet model and benchmark ARIMA model.

Performance of the system over the entire back-testing period and across the selected country of origin to country of destination dyads and the related asylum flows. For each dyad, the relative and absolute average forecasting errors are shown. A simple traffic light system is used for quick inspection of general performance results.

Traffic-light system legend. Colours indicate general forecasting performance, ranging from very good (light green), to good (dark green), poor (orange) and very poor (red) performance. An additional blue shading denotes those instances in which ARIMA outperforms DynENet.

Supplementary Note 2. Summary performance results on sample of flows

Supplementary Figures 1-10 provide statistics and details about the forecasting performance of the system on selected country-to-country asylum flows. The sample flows were selected to provide a diverse set of contexts covering some major destination countries (France, Germany, Greece, Italy, Spain, and the EU+ in the aggregate); some major countries of origin, from different global regions (Middle East, Asia, Africa, South America); relatively more (e.g., Nigeria to Italy) and less (e.g., Syria to Greece) volatile flows. Moreover, the associated forecasts range between very good (Figures S3, S4, S6 and S7), good (Figures S2 and S5), poor (Figure S1), and very poor (Figures S8 and S9).

a

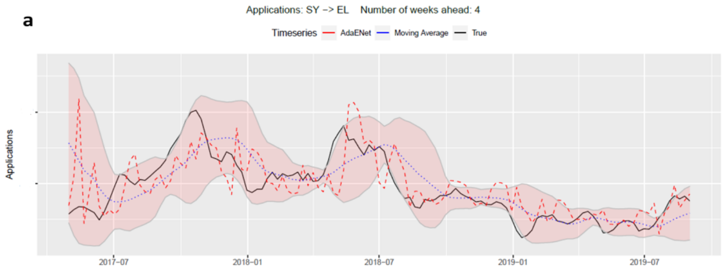

d

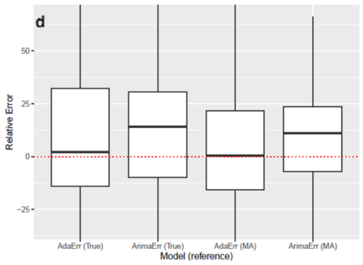

b

type	ahead	minT	maxT	Q1	Q3	IQR	Median	Mean	SD
AdaENet	4	2017−04−30	2019−09−01	−13.9	32.3	46.2	2.0	13.2	44.3
Arima	4	2017−04−30	2019−09−01	−10.0	30.7	40.6	14.0	18.5	42.0
AdaENet (MA)	4	2017−04−30	2019−09−01	−15.7	21.5	37.3	0.4	4.5	29.5
Arima (MA)	4	2017−04−30	2019−09−01	−7.0	23.7	30.7	11.1	9.1	23.7

c

type	ahead	minT	maxT	Q1	Q3	IQR	Median	Mean	SD
AdaENet	4	2017−04−30	2019−09−01	−140	221	362	12	31	331
Arima	4	2017−04−30	2019−09−01	−84	250	334	122	80	268
AdaENet (MA)	4	2017−04−30	2019−09−01	−144	173	317	3	−2	287
Arima (MA)	4	2017−04−30	2019−09−01	−67	191	258	83	48	230

e

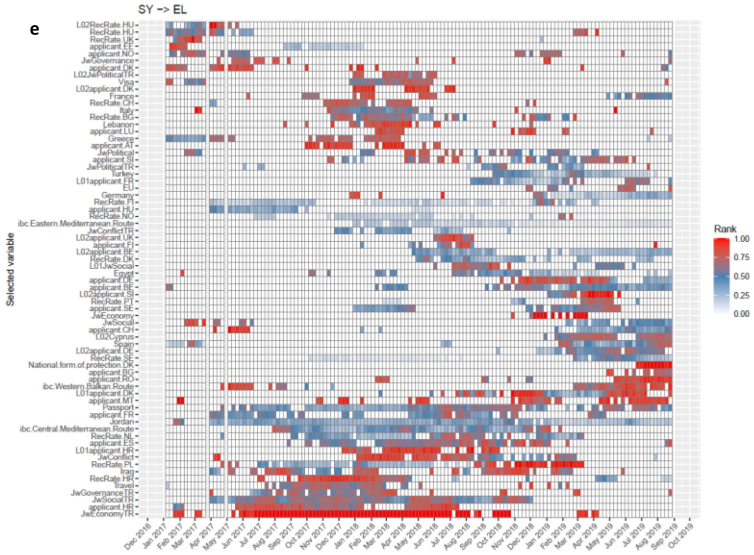

Supplementary Figure S1. Back-testing performance of the system for forecasted applications lodged by Syrians in Greece.

a. The black line shows the actual number of applications lodged by Syrian nationals in Greece. The dotted blue line is the moving average of the process. The red dashed line shows the DynENet 4-week ahead forecast at each time point. The pink shaded area represents a ± 2-standard errors confidence bands around the moving average.

b-d. Summary statistics for the relative error (**b, d**) and for the absolute error (**c**). ARIMA is a benchmark model which is only based on the autocorrelation of the applications timeseries.

e. The factors that impact the forecasting model in the period considered. The model adapts over time, some effects are persistent in the first period, then others become more important. The scale colour only represents the relative importance of the variable. The coloured variables are all included in the model, the others have been dropped by DynENet.

a

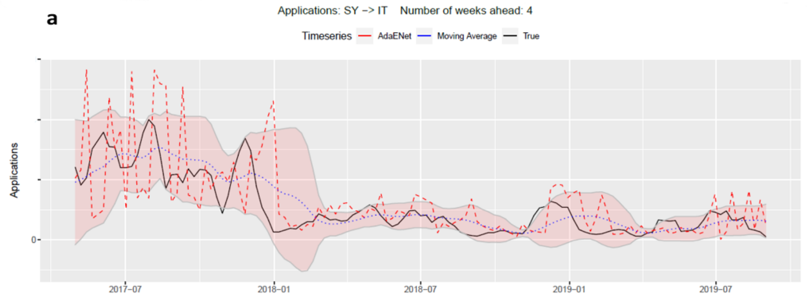

d

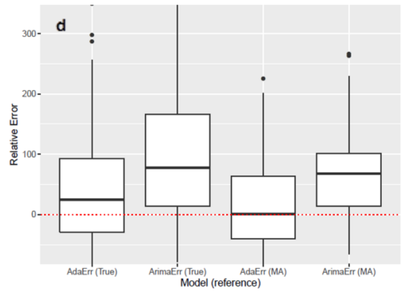

b

type	ahead	minT	maxT	Q1	Q3	IQR	Median	Mean	SD
AdaENet	4	2017-04-30	2019-09-01	-28.8	93.2	122.1	24.7	75.2	208.5
Arima	4	2017-04-30	2019-09-01	13.9	166.1	152.2	77.5	131.6	183.8
AdaENet (MA)	4	2017-04-30	2019-09-01	-40.3	63.7	103.9	1.4	18.3	69.4
Arima (MA)	4	2017-04-30	2019-09-01	13.7	101.8	88.1	68.3	65.5	69.5

c

type	ahead	minT	maxT	Q1	Q3	IQR	Median	Mean	SD
AdaENet	4	2017-04-30	2019-09-01	-9	29	39	6	11	56
Arima	4	2017-04-30	2019-09-01	6	45	39	20	24	45
AdaENet (MA)	4	2017-04-30	2019-09-01	-11	28	39	1	7	47
Arima (MA)	4	2017-04-30	2019-09-01	6	36	30	22	20	34

e

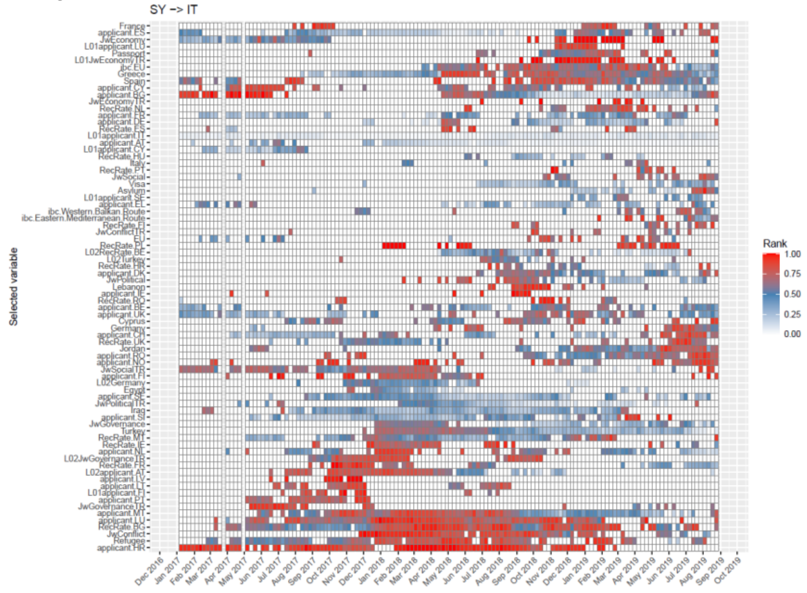

Supplementary Figure S2. Back-testing performance of the system for forecasted applications by Syrians in Italy.

a. The black line shows the actual number of applications lodged by Syrian nationals in Italy. The dotted blue line is the moving average of the process. The red dashed line shows the DynENet 4-week ahead forecast at each time point. The pink shaded area represents a ± 2-standard errors confidence bands around the moving average.

b-d. Summary statistics for the relative error (**b, d**) and for the absolute error (**c**). ARIMA is a benchmark model which is only based on the autocorrelation of the applications timeseries.

e. The factors that impact the forecasting model in the period considered. The model adapts over time, some effects are persistent in the first period, then others become more important. The scale colour only represents the relative importance of the variable. The coloured variables are all included in the model, the others have been dropped by DynENet.

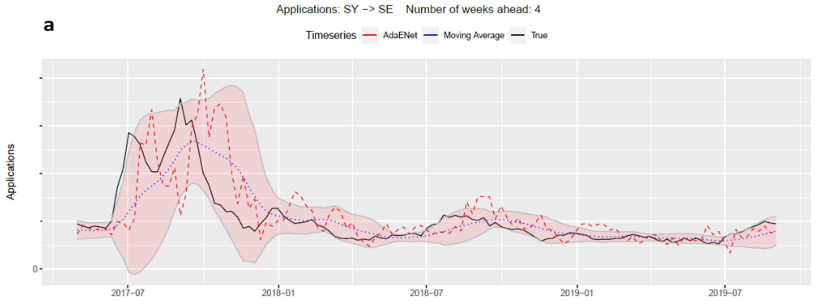

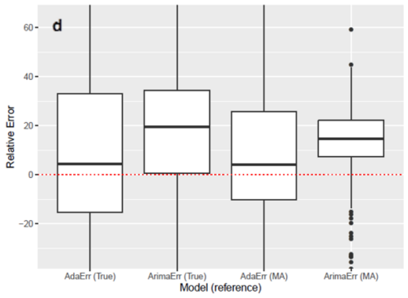

b

type	ahead	minT	maxT	Q1	Q3	IQR	Median	Mean	SD
AdaENet	4	2017-04-30	2019-09-01	-15.3	33.2	48.5	4.3	13.7	43.0
Arima	4	2017-04-30	2019-09-01	0.6	34.4	33.8	19.5	18.2	30.0
AdaENet (MA)	4	2017-04-30	2019-09-01	-10.1	25.9	36.0	4.3	7.8	26.4
Arima (MA)	4	2017-04-30	2019-09-01	7.4	22.2	14.8	14.8	14.4	24.0

c

type	ahead	minT	maxT	Q1	Q3	IQR	Median	Mean	SD
AdaENet	4	2017-04-30	2019-09-01	-29	72	100	10	20	156
Arima	4	2017-04-30	2019-09-01	1	66	64	37	31	122
AdaENet (MA)	4	2017-04-30	2019-09-01	-21	54	76	8	21	97
Arima (MA)	4	2017-04-30	2019-09-01	17	44	28	29	32	95

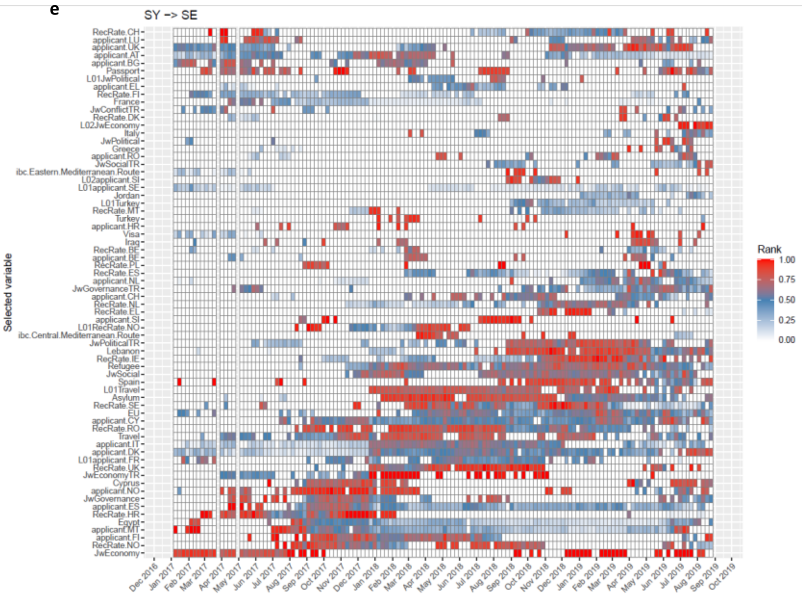

Supplementary Figure S3. Back-testing performance of the system for forecasted applications by Syrians in Sweden.

a. The black line shows the actual number of applications lodged by Syrian nationals in Sweden. The dotted blue line is the moving average of the process. The red dashed line shows the DynENet 4-week ahead forecast at each time point. The pink shaded area represents a ± 2-standard errors confidence bands around the moving average.

b-d. Summary statistics for the relative error (**b, d**) and for the absolute error (**c**). ARIMA is a benchmark model which is only based on the autocorrelation of the applications timeseries.

e. The factors that impact the forecasting model in the period considered. The model adapts over time, some effects are persistent in the first period, then others become more important. The scale colour only represents the relative importance of the variable. The coloured variables are all included in the model, the others have been dropped by DynENet.

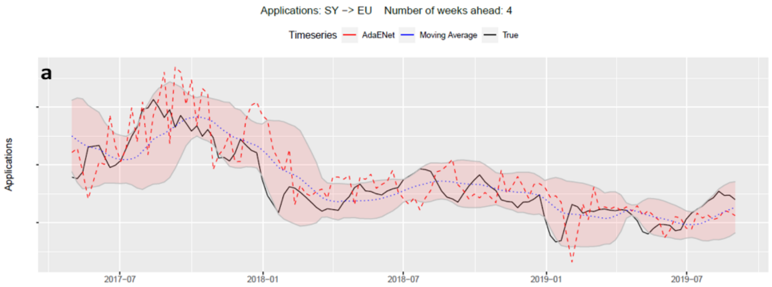

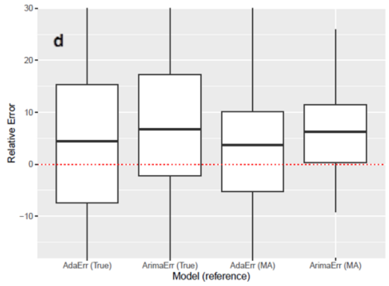

b

type	ahead	minT	maxT	Q1	Q3	IQR	Median	Mean	SD
AdaENet	4	2017-04-30	2019-09-01	-7.4	15.4	22.8	4.4	5.6	16.7
Arima	4	2017-04-30	2019-09-01	-2.2	17.3	19.5	6.8	8.4	16.1
AdaENet (MA)	4	2017-04-30	2019-09-01	-5.2	10.1	15.3	3.7	2.9	12.0
Arima (MA)	4	2017-04-30	2019-09-01	0.3	11.5	11.2	6.2	5.6	10.1

c

type	ahead	minT	maxT	Q1	Q3	IQR	Median	Mean	SD
AdaENet	4	2017-04-30	2019-09-01	-417	804	1221	244	267	877
Arima	4	2017-04-30	2019-09-01	-107	867	974	348	415	806
AdaENet (MA)	4	2017-04-30	2019-09-01	-285	526	812	167	170	714
Arima (MA)	4	2017-04-30	2019-09-01	14	656	642	306	318	612

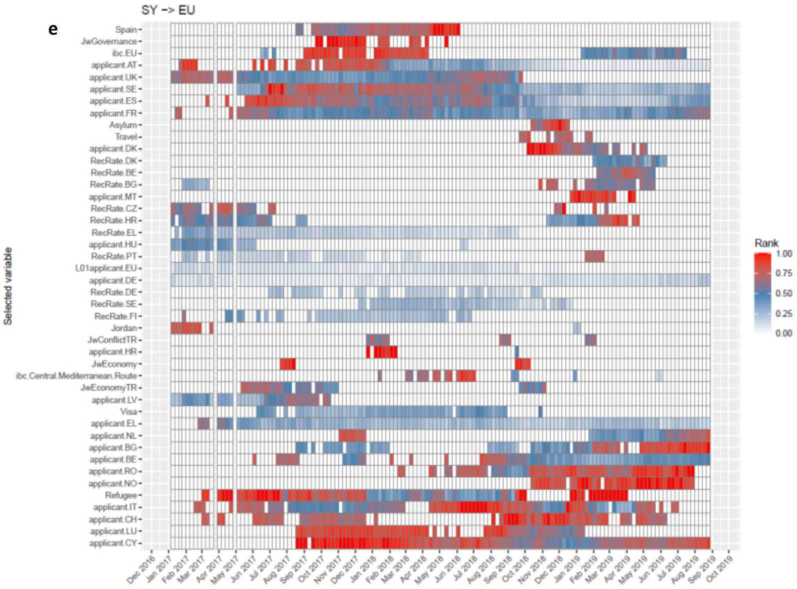

Supplementary Figure S4. Back-testing performance of the system for forecasted applications by Syrians in the EU+.
a. The black line shows the actual number of applications lodged by Syrian nationals in the EU+. The dotted blue line is the moving average of the process. The red dashed line shows the DynENet 4-week ahead forecast at each time point. The pink shaded area represents a ± 2-standard errors confidence bands around the moving average.
b-d. Summary statistics for the relative error (**b, d**) and for the absolute error (**c**). ARIMA is a benchmark model which is only based on the autocorrelation of the applications timeseries.
e. The factors that impact the forecasting model in the period considered. The model adapts over time, some effects are persistent in the first period, then others become more important. The scale colour only represents the relative importance of the variable. The coloured variables are all included in the model, the others have been dropped by DynENet.

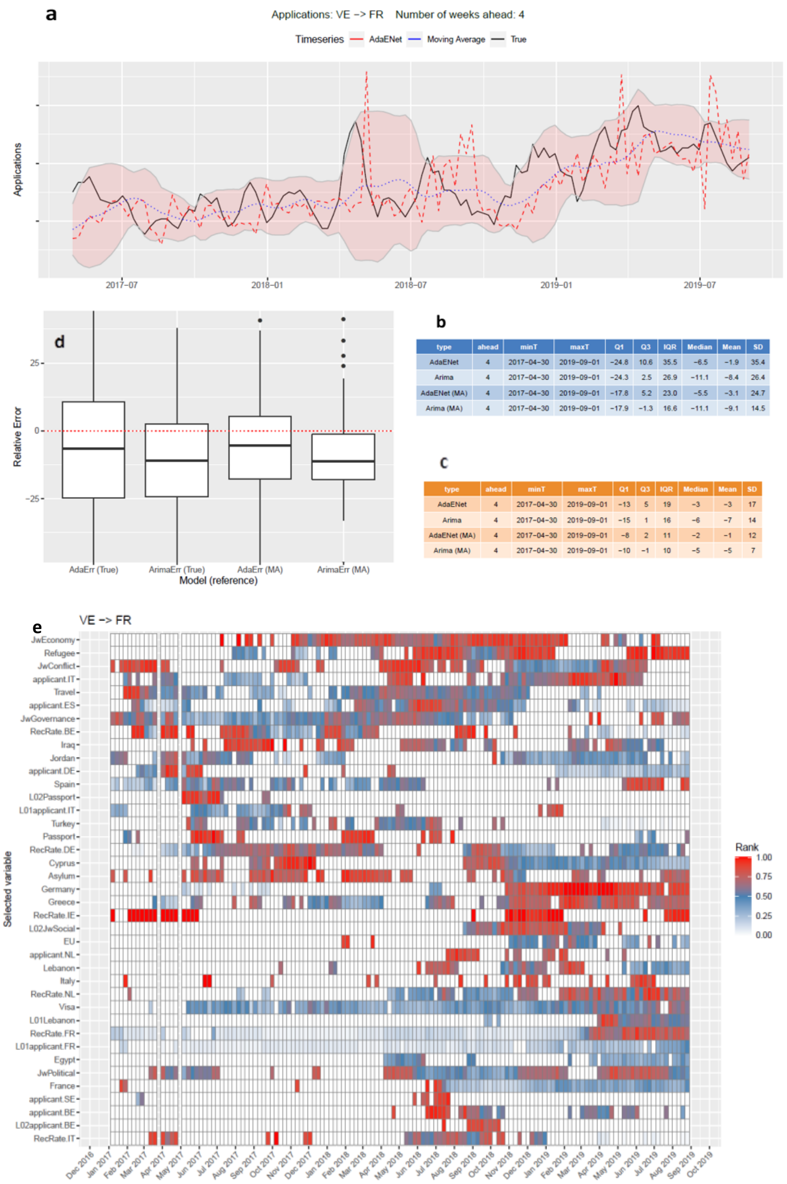

b

type	ahead	minT	maxT	Q1	Q3	IQR	Median	Mean	SD
AdaENet	4	2017-04-30	2019-09-01	-24.8	10.6	35.5	-6.5	-1.9	35.4
Arima	4	2017-04-30	2019-09-01	-24.3	2.5	26.9	-11.1	-8.4	26.4
AdaENet (MA)	4	2017-04-30	2019-09-01	-17.8	5.2	23.0	-5.5	-3.1	24.7
Arima (MA)	4	2017-04-30	2019-09-01	-17.9	-1.3	16.6	-11.1	-9.1	14.5

c

type	ahead	minT	maxT	Q1	Q3	IQR	Median	Mean	SD
AdaENet	4	2017-04-30	2019-09-01	-13	5	19	-3	-3	17
Arima	4	2017-04-30	2019-09-01	-15	1	16	-6	-7	14
AdaENet (MA)	4	2017-04-30	2019-09-01	-8	2	11	-2	-1	12
Arima (MA)	4	2017-04-30	2019-09-01	-10	-1	10	-5	-5	7

Supplementary Figure S5. Back-testing performance of the system for forecasted applications by Venezuelans in France.

a. The black line shows the actual number of applications lodged by Venezuelan nationals in France. The dotted blue line is the moving average of the process. The red dashed line shows the DynENet 4-week ahead forecast at each time point. The pink shaded area represents a ± 2-standard errors confidence bands around the moving average.

b-d. Summary statistics for the relative error (**b, d**) and for the absolute error (**c**). ARIMA is a benchmark model which is only based on the autocorrelation of the applications timeseries.

e. The factors that impact the forecasting model in the period considered. The model adapts over time, some effects are persistent in the first period, then others become more important. The scale colour only represents the relative importance of the variable. The coloured variables are all included in the model, the others have been dropped by DynENet.

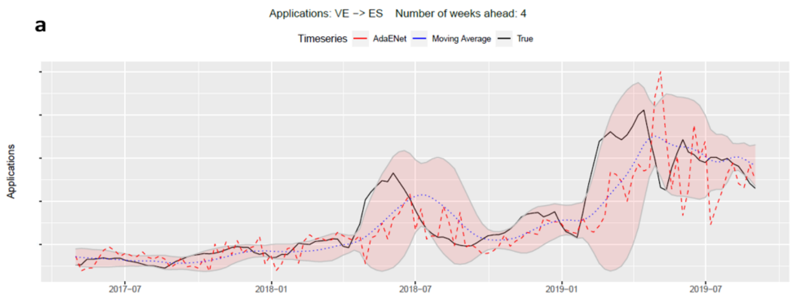

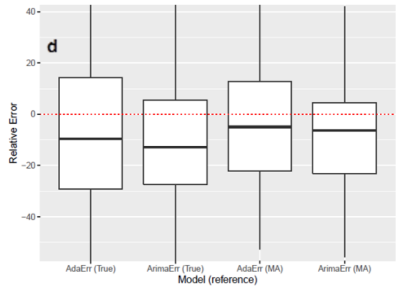

b

type	ahead	minT	maxT	Q1	Q3	IQR	Median	Mean	SD
AdaENet	4	2017-04-30	2019-09-01	-29.3	14.2	43.5	-9.5	-6.0	31.5
Arima	4	2017-04-30	2019-09-01	-27.4	5.5	32.8	-12.9	-13.3	30.4
AdaENet (MA)	4	2017-04-30	2019-09-01	-22.2	12.8	35.0	-4.9	-4.3	24.3
Arima (MA)	4	2017-04-30	2019-09-01	-23.1	4.5	27.6	-6.4	-11.5	25.3

c

type	ahead	minT	maxT	Q1	Q3	IQR	Median	Mean	SD
AdaENet	4	2017-04-30	2019-09-01	-419	137	557	-97	-214	645
Arima	4	2017-04-30	2019-09-01	-388	36	424	-143	-296	666
AdaENet (MA)	4	2017-04-30	2019-09-01	-292	115	407	-72	-124	418
Arima (MA)	4	2017-04-30	2019-09-01	-343	45	388	-71	-206	525

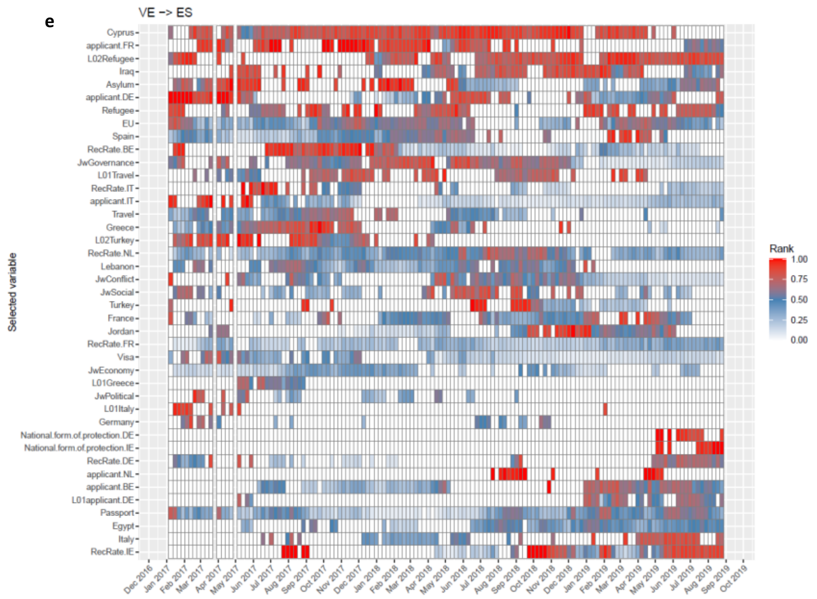

Supplementary Figure S6. Back-testing performance of the system for forecasted applications by Venezuelans in Spain.

a. The black line shows the actual number of applications lodged by Venezuelan nationals in Spain. The dotted blue line is the moving average of the process. The red dashed line shows the DynENet 4-week ahead forecast at each time point. The pink shaded area represents a ± 2-standard errors confidence bands around the moving average.

b-d. Summary statistics for the relative error (**b, d**) and for the absolute error (**c**). ARIMA is a benchmark model which is only based on the autocorrelation of the applications timeseries.

e. The factors that impact the forecasting model in the period considered. The model adapts over time, some effects are persistent in the first period, then others become more important. The scale colour only represents the relative importance of the variable. The coloured variables are all included in the model, the others have been dropped by DynENet.

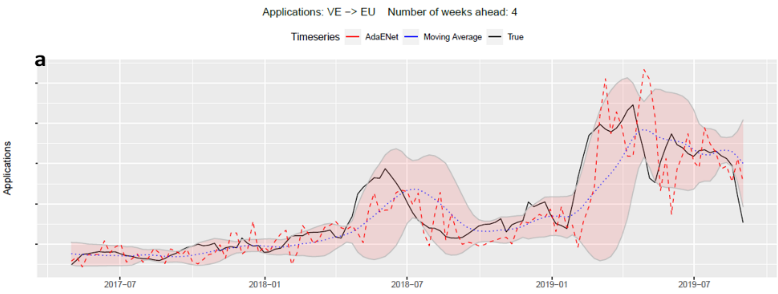

b

type	ahead	minT	maxT	Q1	Q3	IQR	Median	Mean	SD
AdaENet	4	2017-04-30	2019-09-01	−24.7	15.0	39.6	−7.5	−2.9	30.1
Arima	4	2017-04-30	2019-09-01	−26.1	4.1	30.2	−11.1	−11.0	31.1
AdaENet (MA)	4	2017-04-30	2019-09-01	−19.1	14.6	33.7	−3.0	−0.3	26.2
Arima (MA)	4	2017-04-30	2019-09-01	−21.3	5.5	26.8	−4.8	−9.5	24.9

c

type	ahead	minT	maxT	Q1	Q3	IQR	Median	Mean	SD
AdaENet	4	2017-04-30	2019-09-01	−384	196	579	−123	−101	641
Arima	4	2017-04-30	2019-09-01	−410	37	447	−147	−262	700
AdaENet (MA)	4	2017-04-30	2019-09-01	−259	187	446	−40	−11	536
Arima (MA)	4	2017-04-30	2019-09-01	−283	80	364	−58	−171	522

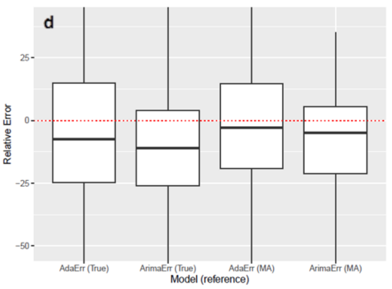

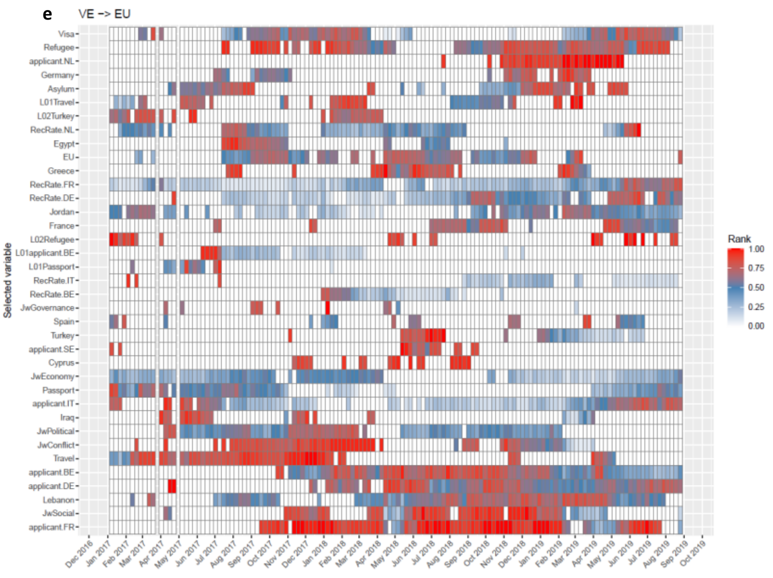

Supplementary Figure S7. Back-testing performance of the system for forecasted applications by Venezuelans in the EU+.

a. The black line shows the actual number of applications lodged by Venezuelan nationals in the EU+. The dotted blue line is the moving average of the process. The red dashed line shows the DynENet 4-week ahead forecast at each time point. The pink shaded area represents a ± 2-standard errors confidence bands around the moving average.

b-d. Summary statistics for the relative error (**b, d**) and for the absolute error (**c**). ARIMA is a benchmark model which is only based on the autocorrelation of the applications timeseries.

e. The factors that impact the forecasting model in the period considered. The model adapts over time, some effects are persistent in the first period, then others become more important. The scale colour only represents the relative importance of the variable. The coloured variables are all included in the model, the others have been dropped by DynENet.

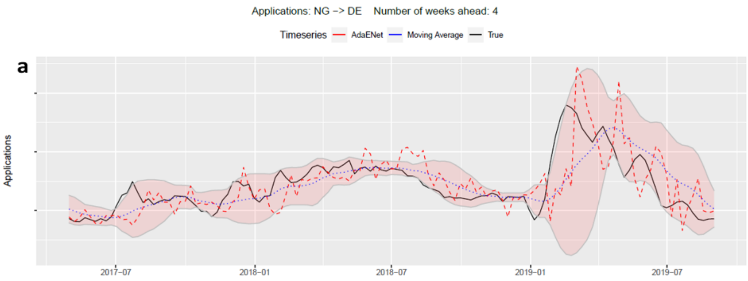

b

type	ahead	minT	maxT	Q1	Q3	IQR	Median	Mean	SD
AdaENet	4	2017-04-30	2019-09-01	-12.2	18.4	30.6	2.4	3.2	26.4
Arima	4	2017-04-30	2019-09-01	-18.5	13.1	31.6	-2.3	-2.0	23.5
AdaENet (MA)	4	2017-04-30	2019-09-01	-10.8	9.1	19.9	-0.9	0.1	19.8
Arima (MA)	4	2017-04-30	2019-09-01	-12.9	0.8	13.7	-4.3	-5.6	11.3

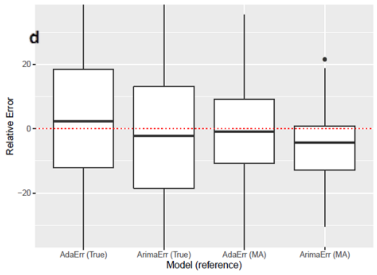

c

type	ahead	minT	maxT	Q1	Q3	IQR	Median	Mean	SD
AdaENet	4	2017-04-30	2019-09-01	-93	119	212	13	4	210
Arima	4	2017-04-30	2019-09-01	-141	78	219	-15	-49	185
AdaENet (MA)	4	2017-04-30	2019-09-01	-67	63	130	-6	3	164
Arima (MA)	4	2017-04-30	2019-09-01	-86	5	91	-32	-49	94

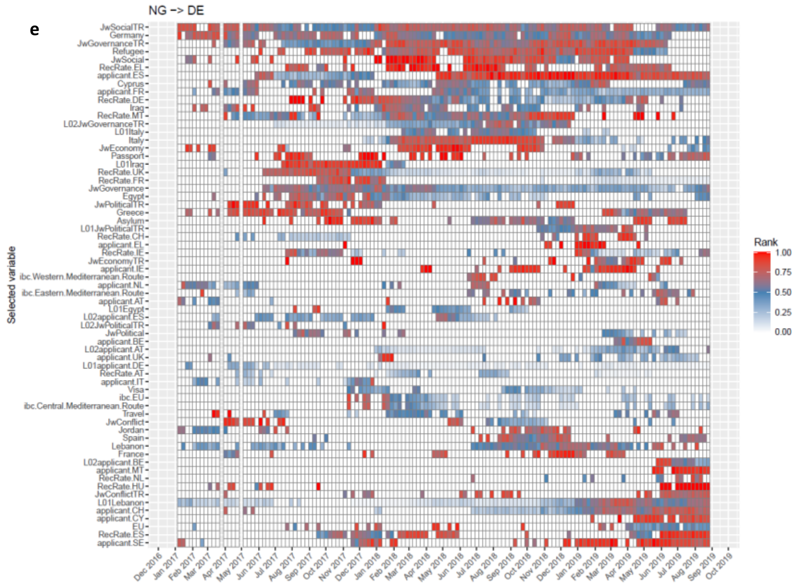

Supplementary Figure S8. Back-testing performance of the system for forecasted applications by Nigerians in Germany.

a. The black line shows the actual number of applications lodged by Nigerian nationals in Germany. The dotted blue line is the moving average of the process. The red dashed line shows the DynENet 4-week ahead forecast at each time point. The pink shaded area represents a ± 2-standard errors confidence bands around the moving average.

b-d. Summary statistics for the relative error (**b, d**) and for the absolute error (**c**). ARIMA is a benchmark model which is only based on the autocorrelation of the applications timeseries.

e. The factors that impact the forecasting model in the period considered. The model adapts over time, some effects are persistent in the first period, then others become more important. The scale colour only represents the relative importance of the variable. The coloured variables are all included in the model, the others have been dropped by DynENet.

a

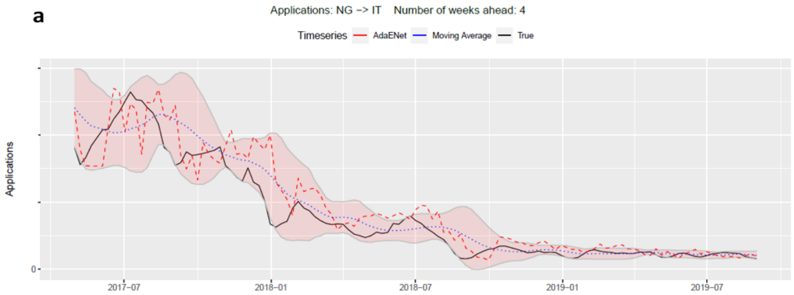

d

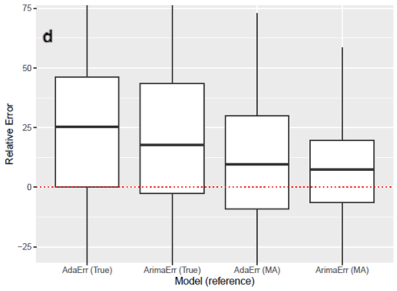

b

type	ahead	minT	maxT	Q1	Q3	IQR	Median	Mean	SD
AdaENet	4	2017-04-30	2019-09-01	0.0	46.3	46.3	25.3	26.9	39.6
Arima	4	2017-04-30	2019-09-01	-2.7	43.6	46.3	17.7	26.4	44.3
AdaENet (MA)	4	2017-04-30	2019-09-01	-9.0	29.8	38.9	9.6	10.1	27.4
Arima (MA)	4	2017-04-30	2019-09-01	-6.5	19.6	26.1	7.3	7.3	19.5

c

type	ahead	minT	maxT	Q1	Q3	IQR	Median	Mean	SD
AdaENet	4	2017-04-30	2019-09-01	-3	261	264	109	134	268
Arima	4	2017-04-30	2019-09-01	-20	226	246	84	99	237
AdaENet (MA)	4	2017-04-30	2019-09-01	-46	163	210	51	38	235
Arima (MA)	4	2017-04-30	2019-09-01	-38	81	120	44	3	160

e

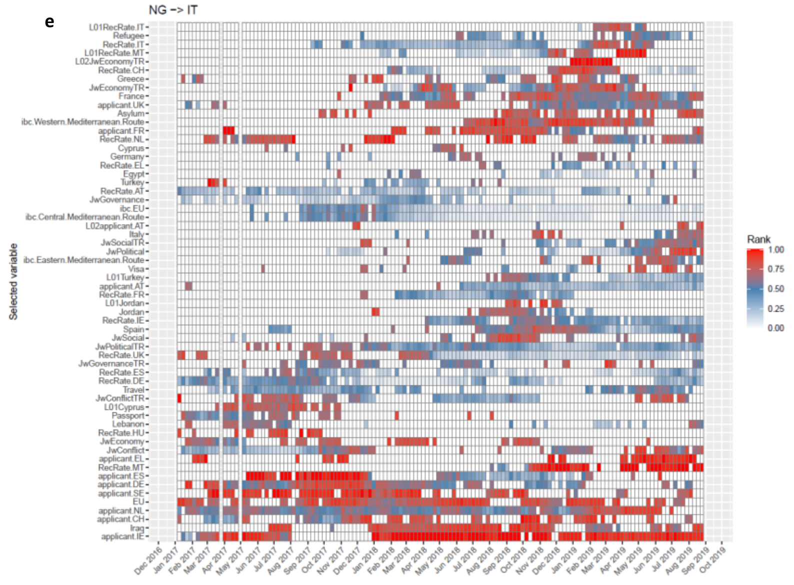

Supplementary Figure S9. Back-testing performance of the system for forecasted applications by Nigerians in Italy.

a. The black line shows the actual number of applications lodged by Nigerian nationals in Italy. The dotted blue line is the moving average of the process. The red dashed line shows the DynENet 4-week ahead forecast at each time point. The pink shaded area represents a ± 2-standard errors confidence bands around the moving average.

b-d. Summary statistics for the relative error (**b, d**) and for the absolute error (**c**). ARIMA is a benchmark model which is only based on the autocorrelation of the applications timeseries.

e. The factors that impact the forecasting model in the period considered. The model adapts over time, some effects are persistent in the first period, then others become more important. The scale colour only represents the relative importance of the variable. The coloured variables are all included in the model, the others have been dropped by DynENet.

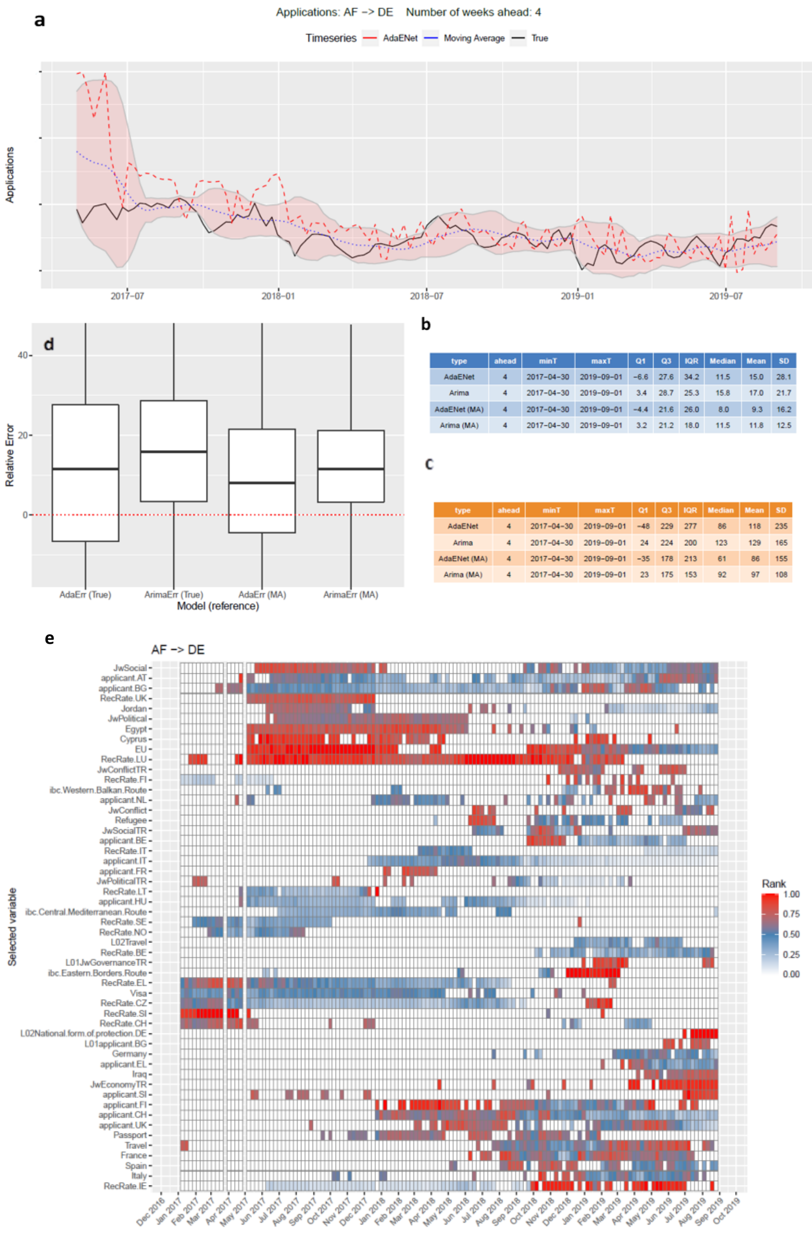

b

type	ahead	minT	maxT	Q1	Q3	IQR	Median	Mean	SD
AdaENet	4	2017-04-30	2019-09-01	-6.6	27.6	34.2	11.5	15.0	28.1
Arima	4	2017-04-30	2019-09-01	3.4	28.7	25.3	15.8	17.0	21.7
AdaENet (MA)	4	2017-04-30	2019-09-01	-4.4	21.6	26.0	8.0	9.3	16.2
Arima (MA)	4	2017-04-30	2019-09-01	3.2	21.2	18.0	11.5	11.8	12.5

c

type	ahead	minT	maxT	Q1	Q3	IQR	Median	Mean	SD
AdaENet	4	2017-04-30	2019-09-01	-48	229	277	86	118	235
Arima	4	2017-04-30	2019-09-01	24	224	200	123	129	165
AdaENet (MA)	4	2017-04-30	2019-09-01	-35	178	213	61	86	155
Arima (MA)	4	2017-04-30	2019-09-01	23	175	153	92	97	108

Supplementary Figure S10. Back-testing performance of the system for forecasted applications by Afghans in Germany.

a. The black line shows the actual number of applications lodged by Afghan nationals in Germany. The dotted blue line is the moving average of the process. The red dashed line shows the DynENet 4-week ahead forecast at each time point. The pink shaded area represents a ± 2-standard errors confidence bands around the moving average.

b-d. Summary statistics for the relative error (**b, d**) and for the absolute error (**c**). ARIMA is a benchmark model which is only based on the autocorrelation of the applications timeseries.

e. The factors that impact the forecasting model in the period considered. The model adapts over time, some effects are persistent in the first period, then others become more important. The scale colour only represents the relative importance of the variable. The coloured variables are all included in the model, the others have been dropped by DynENet.

Supplementary Note 3. Event categories: selection and transformation

Table S2 includes all the event categories within the GDELT-CAMEO event codebook (See GDELT codebook http://data.gdeltproject.org/documentation/GDELT-Event_Codebook-V2.0.pdf and CAMEO scheme http://data.gdeltproject.org/documentation/CAMEO.Manual.1.1b3.pdf), as well as details on the construction of the event indices for the purpose of this study:

a) The events selected as potential drivers of migration (*GDELT code and topic*);

b) The *sign* associated to single events, denoting a potential to generate (+) or constrain (-) international displacements;

c) The grouping factor (*category label*) used to aggregate selected events into the five macro-categories defined for the purposes of this study: conflict, economic events, social unrest, governance-related events, political events;

d) The *strength* of the potential displacement-generation effect associated to single events.

Supplementary Table S2. Event categories within the GDELT-CAMEO event codebook, and details on the construction of the event indices for the purpose of this study.
- The events selected as potential drivers of migration (GDELT code and topic);
- The sign associated to single events, denoting a potential to generate (+) or constrain (-) international displacements;
- The grouping factor (category label) used to aggregate selected events into the five macro-categories defined for the purposes of this study: conflict, economic events, social unrest, governance-related events, political events;
- The strength of the potential displacement-generation effect associated to single events.

GDELT code and topic	Included	Sign	Category label	Strength
110: Disapprove, not specified below	No			
111: Criticize or denounce	No			
112: Accuse, not specified below	No			
0232: Appeal for military aid	Yes	+	Conflict	1
0234: Appeal for military protection or peacekeeping	Yes	+	Conflict	1
0254: Appeal for easing of economic sanctions, boycott, or embargo	Yes	+	Economic	1
0255: Appeal for target to allow international involvement (non-mediation)	Yes	+	Conflict	1

GDELT code and topic	Included	Sign	Category label	Strength
025: Social: Appeal for de-escalation of military engagement	Yes	+	Conflict	1
02 Social: Appeal to others to meet or negotiate	Yes	+	Conflict	1
027: Appeal to others to settle dispute	Yes	+	Conflict	1
028: Appeal to engage in or accept mediation	Yes	+	Conflict	1
0354: Express intent to ease economic sanctions, boycott, or embargo	Yes	-	Economic	1
0355: Express intent to allow international involvement (non-mediation)	Yes	-	Conflict	1
035Social: Express intent to de-escalate military engagement	Yes	-	Conflict	1
03Social: Express intent to meet or negotiate	Yes	-	Conflict	1
037: Express intent to settle dispute	Yes	-	Conflict	1
038: Express intent to accept mediation	Yes	-	Conflict	1
081: Ease administrative sanctions, not specified below	Yes	-	Conflict	1
0871: Declare truce, ceasefire	Yes	-	Conflict	1
0872: Ease military blockade	Yes	-	Conflict	1
0873: Demobilize armed forces	Yes	-	Conflict	2
0874: Retreat or surrender militarily	Yes	-	Conflict	3
093: Investigate military action	Yes	+	Conflict	2
094: Investigate war crimes	Yes	+	Conflict	2
1012: Demand military cooperation	Yes	+	Conflict	1
1014: Demand intelligence cooperation	Yes	+	Conflict	1
1032: Demand military aid	Yes	+	Conflict	1
1034: Demand military protection or peacekeeping	Yes	+	Conflict	1
1054: Demand easing of economic sanctions, boycott, or embargo	Yes	+	Economic	2
1055: Demand that target allows international involvement (non-mediation)	Yes	+	Conflict	1
105Social: Demand de-escalation of military engagement	Yes	+	Conflict	1
10Social: Demand meeting, negotiation	Yes	+	Conflict	1
107: Demand settling of dispute	Yes	+	Conflict	1
108: Demand mediation	Yes	+	Conflict	1
1123: Accuse of aggression	Yes	+	Conflict	1
1124: Accuse of war crimes	Yes	+	Conflict	1
1125: Accuse of espionage, treason	Yes	+	Conflict	1
1244: Refuse to ease economic sanctions, boycott, or embargo	Yes	+	Economic	2
1245: Refuse to allow international involvement (non-mediation)	Yes	+	Conflict	2
124Social: Refuse to de-escalate military engagement	Yes	+	Conflict	2
125: Reject proposal to meet, discuss, or negotiate	Yes	+	Conflict	2

GDELT code and topic	Included	Sign	Category label	Strength
12Social: Reject mediation	Yes	+	Conflict	2
127: Reject plan, agreement to settle dispute	Yes	+	Conflict	2
138: Threaten with military force, not specified below	Yes	+	Conflict	1
1382: Threaten occupation	Yes	+	Conflict	2
1383: Threaten unconventional violence	Yes	+	Conflict	2
1384: Threaten conventional attack	Yes	+	Conflict	2
1385: Threaten attack with WMD	Yes	+	Conflict	2
139: Give ultimatum	Yes	+	Conflict	2
150: Demonstrate military or police power, not specified below	Yes	+	Conflict	2
152: Increase military alert status	Yes	+	Conflict	1
154: Mobilize or increase armed forces	Yes	+	Conflict	2
155: Mobilize or increase cyber-forces	Yes	+	Conflict	2
1Social4: Halt negotiations	Yes	+	Conflict	2
1Social5: Halt mediation	Yes	+	Conflict	1
1SocialSocial: Expel or withdraw, not specified below	Yes	+	Conflict	2
1SocialSocial1: Expel or withdraw peacekeepers	Yes	+	Conflict	2
1SocialSocial2: Expel or withdraw inspectors, observers	Yes	+	Conflict	2
1SocialSocial3: Expel or withdraw aid agencies	Yes	+	Conflict	2
170: Coerce, not specified below	Yes	+	Conflict	2
17Social: Attack cybernetically	Yes	+	Conflict	1
180: Use unconventional violence, not specified below	Yes	+	Conflict	3
190: Use conventional military force, not specified below	Yes	+	Conflict	3
191: Impose blockade, restrict movement	Yes	+	Conflict	2
192: Occupy territory	Yes	+	Conflict	3
193: Fight with small arms and light weapons	Yes	+	Conflict	3
194: Fight with artillery and tanks	Yes	+	Conflict	3
195: Employ aerial weapons, not specified below	Yes	+	Conflict	3
1951: Employ precision-guided aerial munitions	Yes	+	Conflict	3
1952: Employ remotely piloted aerial munitions	Yes	+	Conflict	3
19Social: Violate ceasefire	Yes	+	Conflict	3
200: Use unconventional mass violence, not specified below	Yes	+	Conflict	4
204: Use weapons of mass destruction, not specified below	Yes	+	Conflict	4
2041: Use chemical, biological, or radiological weapons	Yes	+	Conflict	4
2042: Detonate nuclear weapons	Yes	+	Conflict	4
1033: Demand humanitarian aid	Yes	+	Conflict	2

GDELT code and topic	Included	Sign	Category label	Strength
023: Appeal for aid, not specified below	Yes	+	Economic	2
0231: Appeal for economic aid	Yes	+	Economic	2
1011: Demand economic cooperation	Yes	+	Economic	2
103: Demand material aid, not specified below	Yes	+	Economic	2
1031: Demand economic aid	Yes	+	Economic	2
024: Appeal for political reform, not specified below	Yes	+	Governance	1
0241: Appeal for change in leadership	Yes	+	Governance	1
0244: Appeal for change in institutions, regime	Yes	+	Governance	1
034: Express intent to institute political reform, not specified below	Yes	-	Governance	1
0341: Express intent to change leadership	Yes	-	Governance	1
0342: Express intent to change policy	Yes	-	Governance	1
0344: Express intent to change institutions, regime	Yes	-	Governance	1
035: Express intent to yield, not specified below	Yes	-	Governance	1
083: Accede to requests or demands for political reform, not specified below	Yes	-	Governance	2
0831: Accede to demands for change in leadership	Yes	-	Governance	2
0832: Accede to demands for change in policy	Yes	-	Governance	2
0834: Accede to demands for change in institutions, regime	Yes	-	Governance	2
091: Investigate crime, corruption	Yes	+	Governance	1
104: Demand political reform, not specified below	Yes	+	Governance	1
1041: Demand change in leadership	Yes	+	Governance	1
1042: Demand policy change	Yes	+	Governance	1
1044: Demand change in institutions, regime	Yes	-	Governance	1
105: Demand that target yields, not specified below	Yes	+	Governance	1
1121: Accuse of crime, corruption	Yes	+	Governance	1
123: Reject request or demand for political reform, not specified below	Yes	+	Governance	1
1231: Reject request for change in leadership	Yes	+	Governance	1
1232: Reject request for policy change	Yes	+	Governance	1
1234: Reject request for change in institutions, regime	Yes	+	Governance	1
124: Refuse to yield, not specified below	Yes	+	Governance	1
1241: Refuse to ease administrative sanctions	Yes	+	Governance	1
128: Defy norms, law	Yes	+	Governance	1
0243: Appeal for rights	Yes	+	Political	1
0251: Appeal for easing of administrative sanctions	Yes	+	Political	1
0253: Appeal for release of persons or property	Yes	+	Political	1
0343: Express intent to provide rights	Yes	-	Political	1
0351: Express intent to ease administrative sanctions	Yes	-	Political	1
0353: Express intent to release persons or property	Yes	-	Political	1
075: Grant asylum	Yes	-	Political	2

GDELT code and topic	Included	Sign	Category label	Streng th
0811: Ease restrictions on political freedoms	Yes	-	Political	2
0812: Ease ban on political parties or politicians	Yes	-	Political	2
0813: Ease curfew	Yes	-	Political	2
0814: Ease state of emergency or martial law	Yes	-	Political	2
0833: Accede to demands for rights	Yes	-	Political	2
092: Investigate human rights abuses	Yes	+	Political	1
1043: Demand rights	Yes	+	Political	1
1051: Demand easing of administrative sanctions	Yes	+	Political	1
1053: Demand release of persons or property	Yes	+	Political	1
1122: Accuse of human rights abuses	Yes	+	Political	1
1233: Reject request for rights	Yes	+	Political	1
1243: Refuse to release persons or property	Yes	+	Political	2
1322: Threaten to ban political parties or politicians	Yes	+	Political	1
1323: Threaten to impose curfew	Yes	+	Political	1
1324: Threaten to impose state of emergency or martial law	Yes	+	Political	1
137: Threaten with repression	Yes	+	Political	1
151: Increase police alert status	Yes	+	Political	2
153: Mobilize or increase police power	Yes	+	Political	2
1711: Confiscate property	Yes	+	Political	3
172: Impose administrative sanctions, not specified below	Yes	+	Political	3
1721: Impose restrictions on political freedoms	Yes	+	Political	3
1722: Ban political parties or politicians	Yes	+	Political	3
1723: Impose curfew	Yes	+	Political	3
1724: Impose state of emergency or martial law	Yes	+	Political	3
173: Arrest, detain, or charge with legal action	Yes	+	Political	3
174: Expel or deport individuals	Yes	+	Political	3
175: Use tactics of violent repression	Yes	+	Political	3
181: Abduct, hijack, or take hostage	Yes	+	Political	3
1822: Torture	Yes	+	Political	3
1823: Kill by physical assault	Yes	+	Political	3
201: Engage in mass expulsion	Yes	+	Conflict	3
202: Engage in mass killings	Yes	+	Conflict	3
203: Engage in ethnic cleansing	Yes	+	Conflict	3
0233: Appeal for humanitarian aid	Yes	+	Conflict	1
0252: Appeal for easing of political dissent	Yes	+	Social	1
0352: Express intent to ease popular dissent	Yes	-	Social	1
082: Ease political dissent	Yes	-	Social	2
084: Return, release, not specified below	Yes	-	Political	2
0841: Return, release person(s)	Yes	-	Political	2
0842: Return, release property	Yes	-	Political	2

GDELT code and topic	Included	Sign	Category label	Streng th
1052: Demand easing of political dissent	Yes	+	Political	1
113: Rally opposition against	Yes	+	Social	2
1242: Refuse to ease popular dissent	Yes	+	Social	2
133: Threaten with political dissent, protest	Yes	+	Social	1
1381: Threaten blockade	Yes	+	Social	1
140: Engage in political dissent, not specified below	Yes	+	Social	1
141: Demonstrate or rally, not specified below	Yes	+	Social	1
1411: Demonstrate for leadership change	Yes	+	Social	2
1412: Demonstrate for policy change	Yes	+	Social	1
1413: Demonstrate for rights	Yes	+	Social	1
1414: Demonstrate for change in institutions, regime	Yes	+	Social	2
142: Conduct hunger strike, not specified below	Yes	+	Social	1
1421: Conduct hunger strike for leadership change	Yes	+	Social	2
1422: Conduct hunger strike for policy change	Yes	+	Social	1
1423: Conduct hunger strike for rights	Yes	+	Social	1
1424: Conduct hunger strike for change in institutions, regime	Yes	+	Social	2
143: Conduct strike or boycott, not specified below	Yes	+	Social	1
1431: Conduct strike or boycott for leadership change	Yes	+	Social	2
1432: Conduct strike or boycott for policy change	Yes	+	Social	1
1433: Conduct strike or boycott for rights	Yes	+	Social	1
1434: Conduct strike or boycott for change in institutions, regime	Yes	+	Social	2
144: Obstruct passage, block, not specified below	Yes	+	Social	1
1441: Obstruct passage to demand leadership change	Yes	+	Social	2
1442: Obstruct passage to demand policy change	Yes	+	Social	1
1443: Obstruct passage to demand rights	Yes	+	Social	1
1444: Obstruct passage to demand change in institutions, regime	Yes	+	Social	2
145: Protest violently, riot, not specified below	Yes	+	Social	2
1451: Engage in violent protest for leadership change	Yes	+	Social	3
1452: Engage in violent protest for policy change	Yes	+	Social	3
1453: Engage in violent protest for rights	Yes	+	Social	3
1454: Engage in violent protest for change in institutions, regime	Yes	+	Social	3
171: Seize or damage property, not specified below	Yes	+	Social	1
1712: Destroy property	Yes	+	Social	1
182: Physically assault, not specified below	Yes	+	Social	2
1821: Sexually assault	Yes	+	Social	1
183: Conduct suicide, car, or other non-military bombing, not specified below	Yes	+	Social	3
1831: Carry out suicide bombing	Yes	+	Social	3

GDELT code and topic	Included	Sign	Category label	Streng th
1832: Carry out vehicular bombing	Yes	+	Social	3
1833: Carry out roadside bombing	Yes	+	Social	3
1834: Carry out location bombing	Yes	+	Social	1
184: Use as human shield	Yes	+	Social	3
185: Attempt to assassinate	Yes	+	Social	2
18Social: Assassinate	Yes	+	Social	2
012: Make pessimistic comment	No			
015: Acknowledge or claim responsibility	No			
0312: Express intent to cooperate militarily	No			
07: PROVIDE AID	Yes	-	Conflict	1
070: Provide aid, not specified below	Yes	+	Conflict	2
071: Provide economic aid	Yes	-	Economic	2
072: Provide military aid	Yes	+	Conflict	2
073: Provide humanitarian aid	Yes	-	Conflict	2
074: Provide military protection or peacekeeping	Yes	-	Conflict	2
085: Ease economic sanctions, boycott, embargo	Yes	-	Conflict	2
08Social: Allow international involvement, not specified below	Yes	-	Conflict	2
08Social1: Receive deployment of peacekeepers	Yes	-	Conflict	2
08Social2: Receive inspectors	Yes	-	Conflict	1
08Social3: Allow humanitarian access	Yes	-	Conflict	2
12: REJECT	No			
13: THREATEN	Yes	+	Conflict	1
1Social: REDUCE RELATIONS	Yes	+	Conflict	1
01: MAKE PUBLIC STATEMENT	No			
010: Make statement, not specified below	No			
011: Decline comment	No			
013: Make optimistic comment	No			
014: Consider policy option	No			
01Social: Deny responsibility	No			
017: Engage in symbolic act	No			
018: Make empathetic comment	No			
019: Express accord	No			
02: APPEAL	No			
020: Make an appeal or request, not specified below	No			
021: Appeal for material cooperation, not specified below	No			
0211: Appeal for economic cooperation	Yes	+	Economic	1
0212: Appeal for military cooperation	Yes	+	Conflict	1
0213: Appeal for judicial cooperation	No			
0214: Appeal for intelligence	No			

GDELT code and topic	Included	Sign	Category label	Strength
022: Appeal for diplomatic cooperation (such as policy support)	No			
0242: Appeal for policy change	No			
025: Appeal to yield, not specified below	No			
03: EXPRESS INTENT TO COOPERATE	No			
030: Express intent to cooperate, not specified below	No			
031: Express intent to engage in material cooperation, not specified below	No			
0311: Express intent to cooperate economically	Yes	-	Economic	1
0313: Express intent to cooperate on judicial matters	No			
0314: Express intent to cooperate on intelligence	No			
032: Express intent to engage in diplomatic cooperation (such as policy support)	Yes	-	Conflict	1
033: Express intent to provide material aid, not specified below	Yes	-	Conflict	1
0331: Express intent to provide economic aid	Yes	-	Economic	1
0332: Express intent to provide military aid	Yes	+	Conflict	1
0333: Express intent to provide humanitarian aid	Yes	-	Conflict	1
0334: Express intent to provide military protection or peacekeeping	Yes	-	Conflict	1
039: Express intent to mediate	Yes	-	Conflict	1
04: CONSULT	No			
040: Consult, not specified below	No			
041: Discuss by telephone	No			
042: Make a visit	No			
043: Host a visit	No			
044: Meet at a "third" location	No			
045: Mediate	No			
04Social: Engage in negotiation	Yes	-	Conflict	2
05: ENGAGE IN DIPLOMATIC COOPERATION	No			
050: Engage in diplomatic cooperation, not specified below	Yes	-	Conflict	2
051: Praise or endorse	No			
052: Defend verbally	No			
053: Rally support on behalf of	No			
054: Grant diplomatic recognition	Yes	-	Conflict	1
055: Apologize	No			
05Social: Forgive	No			
057: Sign formal agreement	No			
0Social: ENGAGE IN MATERIAL COOPERATION	No			
0Social0: Engage in material cooperation, not specified below	No			

GDELT code and topic	Included	Sign	Category label	Strength
0Social1: Cooperate economically	Yes	-	Economic	2
0Social2: Cooperate militarily	Yes	+	Conflict	2
0Social3: Engage in judicial cooperation	No			
0Social4: Share intelligence or information	No			
08: YIELD	No			
080: Yield, not specified below	No			
087: De-escalate military engagement	Yes	-	Conflict	3
09: INVESTIGATE	No			
090: Investigate, not specified below	No			
10: DEMAND	No			
100: Demand, not specified below	No			
101: Demand material cooperation, not specified below	No			
1013: Demand judicial cooperation	No			
102: Demand diplomatic cooperation (such as policy support)	No			
11: DISAPPROVE	No			
114: Complain officially	No			
115: Bring lawsuit against	No			
11Social: Find guilty or liable (legally)	No			
120: Reject, not specified below	No			
121: Reject material cooperation	No			
1211: Reject economic cooperation	Yes	+	Economic	1
1212: Reject military cooperation	Yes	+	Conflict	1
122: Reject request or demand for material aid, not specified below	Yes	+	Conflict	1
1221: Reject request for economic aid	Yes	+	Conflict	1
1222: Reject request for military aid	Yes	+	Conflict	1
1223: Reject request for humanitarian aid	Yes	+	Conflict	1
1224: Reject request for military protection or peacekeeping	Yes	+	Conflict	1
129: Veto	No			
130: Threaten, not specified below	No			
131: Threaten non-force, not specified below	No			
1311: Threaten to reduce or stop aid	No			
1312: Threaten with sanctions, boycott, embargo	No			
1313: Threaten to reduce or break relations	No			
132: Threaten with administrative sanctions, not specified below	No			
1321: Threaten with restrictions on political freedoms	No			
134: Threaten to halt negotiations	No			
135: Threaten to halt mediation	No			

GDELT code and topic	Included	Sign	Category label	Strength
13Social: Threaten to halt international involvement (non-mediation)	No			
14: PROTEST	Yes	+	Social	1
15: EXHIBIT FORCE POSTURE	Yes	+	Conflict	1
1Social0: Reduce relations, not specified below	No			
1Social1: Reduce or break diplomatic relations	No			
1Social2: Reduce or stop material aid, not specified below	Yes	+	Conflict	2
1Social21: Reduce or stop economic assistance	Yes	+	Economic	2
1Social22: Reduce or stop military assistance	Yes	+	Conflict	2
1Social23: Reduce or stop humanitarian assistance	Yes	+	Conflict	2
1Social3: Impose embargo, boycott, or sanctions	Yes	+	Conflict	2
17: COERCE	No			
18: ASSAULT	No			
19: FIGHT	No			
20: USE UNCONVENTIONAL MASS VIOLENCE	No			
